# Elastic property and fracture mechanics of lateral branch-branch junctions in cacti: A case study of *Opuntia ficus-indica* and *Cylindropuntia bigelovii*

**DOI:** 10.3389/fpls.2022.950860

**Published:** 2022-09-27

**Authors:** Max D. Mylo, Anna Hoppe, Lars Pastewka, Thomas Speck, Olga Speck

**Affiliations:** ^1^Plant Biomechanics Group, Botanic Garden Freiburg, University of Freiburg, Freiburg im Breisgau, Germany; ^2^Cluster of Excellence livMatS, FIT–Freiburg Center for Interactive Materials and Bioinspired Technologies, Freiburg im Breisgau, Germany; ^3^Department of Microsystems Engineering, University of Freiburg, Freiburg im Breisgau, Germany

**Keywords:** abscission, biomechanics, digital image correlation, fracture modes, finite element analysis, geometric model, Opuntioideae, tensile testing

## Abstract

Species with various reproductive modes accompanied by different mechanical properties of their (lateral) branch-branch junctions have evolved in the cactus subfamily Opuntioideae. Older branches of *Opuntia ficus-indica* with fracture-resistant junctions often bear flowers and fruits for sexual reproduction, whereas younger branches break off easily and provide offshoots for vegetative propagation. *Cylindropuntia bigelovii* plants are known for their vegetative reproduction *via* easily detachable branches that can establish themselves as offshoots. We characterized the elastic and fracture behaviors of these lateral junctions by tensile testing and analyzed local strains during loading. Additionally, we carried out finite element analyses to quantify the influence of five relevant tissue layers on joint elastic behavior. Our fracture analysis revealed various fracture modes: (i) most young samples of *Opuntia ficus-indica* failed directly at the junction and had smooth fracture surfaces, and relative fracture strain was on median 4% of the total strain; (ii) most older samples of *Opuntia ficus-indica* failed at the adjacent branch and exhibited rough fracture surfaces, and relative fracture strain was on median 47%; (iii) most samples of *Cylindropuntia bigelovii* abscised directly at the junction and exhibited cup and cone surfaces, and relative fracture strain was on median 28%. Various geometric and mechanical properties such as junction area, fracture energy, and tensile strength were analyzed with respect to significant differences between species and age of sample. Interestingly, the abscission of lateral branches naturally triggered by wind, passing animals, or vibration showed the following differences in maximum force: 153 N (older *Opuntia ficus-indica*), 51 N (young *Opuntia ficus-indica*), and 14 N (*Cylindropuntia bigelovii*).

## Introduction

Cacti are popular examples of xerophytes that are adapted to survive in environments with little water. Their xeromorphic adaptations include aspects of their morphology, anatomy, physiology, and reproduction (Rebman and Pinkava, 2001). Opuntioideae is the second largest subfamily of the Cactaceae family (Griffith and Porter, 2009), comprising over 300 species, with the tribes Opuntieae and Cylindropuntieae being the most prominent species (Guerrero et al., 2019). All Opuntioideae members are united by their general growth habit with chained branches connected by junctions having a pronounced constriction, small barbed and deciduous spines (so-called “glochids”) growing out of areoles, and further xeromorphic adaptations for efficient water use (Benson, 1982; Gibson and Nobel, 1986). The last mentioned includes a multilayered hypodermis, a large number of mucilage glands and oxalate crystals, and branches with geometries that allow rapid water storage (Nobel, 2002). The branches appear flat with an oval cross-section (especially in the genus *Opuntia*, also called “cladodes”) or are cylindrical (especially in the genus *Cylindropuntia*), and in the vast majority of species, their leaves are shed at a very young stage (Benson, 1982).

However, not only the leaves tend to fall off quickly. In some species such as *Cylindropuntia bigelovii* (also known as the “teddy bear cholla” or “jumping cholla”), the lateral branches abscise under a slight mechanical force of less than 10 N (Bobich and Nobel, 2001b). A small fracture area (Bobich and Nobel, 2001b; Mylo et al., 2021a), a good self-sealing capability (Mylo et al., 2020), and a high rooting and establishment rate of the detached branches (Bobich and Nobel, 2001b; Evans et al., 2004) allow a large number of new individuals to develop from these offshoots by vegetative propagation. The spines of *C. bigelovii* possess retrorse-shaped barbs allowing severed branches to attach to the fur or skin of passing vertebrates and thus facilitating dispersal of the offshoots over greater distances (Gibson and Nobel, 1986; Crofts and Anderson, 2018). Although most species of Opuntioideae exhibit a shrubby habit, some *Opuntia* species have a tree-like growth form. For these species, vegetative propagation plays a minor role, and sexual reproduction *via* fruits and seeds is predominant (Benson, 1982; Gibson and Nobel, 1986). Branch-branch junctions that can withstand higher mechanical forces without fracture are prerequisites for such tree-like cacti. One of the best-known representatives of *Opuntias* is *O. ficus-indica*, which reaches a height of up to 5 m and is known for its edible fruits (Reyes-Agüero et al., 2006).

The analysis of the connections of two plant organs with distinct geometric taper and different mechanical properties in closely related species is a very intriguing question not only from an ecological point of view but also from a biomechanical perspective, which has been analyzed in the past using various methodological approaches. Finite element analyses of ramification models have demonstrated that the constrictions between the stem and branches of columnar cacti are not a structural weakness, because load adaptation takes place by fine-tuning of fiber orientations in the cactus wood depending on the stresses and stress directions experienced by the branches (Schwager et al., 2013). In contrast, bending tests carried out on *O. ficus-indica* with cladodes free to flex compared with samples with branch-branch junctions fixed in space have revealed that the junctions are the mechanical weak points in this cactus (Nobel and Meyer, 1991). Field experiments have shown that the mechanical stability of junctions of several *Opuntia* species varies considerably under bending load. Bobich and Nobel (2001a) report that in *O. ficus-indica*, much higher forces are required to bend the junction to a certain deflection angle than that needed in the closely related species *O. occidentalis*, which is known for its vegetative propagation, and *O. littoralis*. In addition, for all three analyzed species, stiffening from the youngest lateral junction to the older, more basal junctions has been observed, with a markedly highest relative increase in stiffness for *O. ficus-indica*. However, by normalizing the stiffness in the junction area, no differences are found for the elastic modulus between the lateral and the more basal junctions (Bobich and Nobel, 2002). The orientation of the force application, i.e., parallel or orthogonal to the cladode surface, has no influence on junction stiffness. In a further field study with a similar bending device, Bobich and Nobel (2001b) have shown that lower forces (between 2 and 8 N) are necessary in *C. bigelovii* to cause failure of the lateral junctions compared with its *Opuntia* relatives (between 40 and 100 N for *O. ficus-indica*; Bobich and Nobel, 2002). In addition, its junctions fail at a markedly lower angular deflection compared with the three other *Cylindropuntia* species tested, and it exhibits the highest root formation rate of detached branches.

A comparison of the anatomy and morphology of the lateral branch-branch junctions of *O. ficus-indica* and *C. bigelovii* (Mylo et al., 2021a) has revealed some similarities but also some dissimilarities. Commonalities are the pronounced taper of the branch cross-section toward the junction by more than 90%, the net-like arrangement of the branch vascular system that converges within the junction, and the change in parenchymatic cells (toward smaller cells with thicker cell walls) and vascular bundles (reduced number of wide-band tracheids) in the junction region. However, the distinct differences include the absolute and relative size of the junction area, which is notably smaller in *C. bigelovii*. Moreover, in *O. ficus-indica*, a collar-like peridermal covering on the epidermis is evident for lateral junctions, whereas in *C. bigelovii*, the covering is only established at more basal and older branch-branch junctions. The mechanical properties tested under tensile loading until failure has revealed differences between isolated tissues of the two species. The elastic modulus and strength of vascular fibers are markedly higher in *O. ficus-indica*. The dermal and underlying tissues, consisting of a uniseriate epidermis and a multi-layered hypodermis, differ little between the two species. However, periderm accumulation on the dermal tissue of older *O. ficus-indica* results in an approximately ten-fold increase in the elastic modulus (Mylo et al., 2021a). The mechanical characterization of the cacti junctions that we present here likewise distinguishes between elastic behavior as an ability to resist external tensile forces and to return to the original state, and fracture behavior as an ability to withstand tensile forces without failure.

Finite element (FE) simulation represents a powerful tool for providing detailed information about biological material systems based on the imported data of its geometric and material properties. In contrast to experiments on plant samples, simulations enable experiments to be performed in which only one variable is changed. Furthermore, scenarios can be simulated in which geometry, shape, size, and material properties are changed in a way that is not found in plants in this combination (Wolff-Vorbeck et al., 2019, 2021). In the biomechanical analysis of columnar cacti, i.e., FE methods have been used to illustrate that their branch-stem junctions can distribute stresses under bending load over larger areas by slight “biomimetic” form optimization (Mattheck, 1998) and thus avoid local stress peaks (Schwager et al., 2013).

The aim of this study is to characterize the elastic and fracture behaviors of the lateral branch-branch junctions of selected species of Opuntioideae by means of tensile tests until break. We have investigated young fracture-prone junctions of *O. ficus-indica*, older fracture-resistant *O. ficus-indica* junctions, and junctions of *C. bigelovii* that is known for their notably fracture-prone junctions. In the context of this study, we have investigated the following three main scientific questions: (i) what are the elastic properties of junctions under tensile loading, and how can the interpretation based on experimental form-structure-function analysis be supported by FE analyses? (ii) What is the load-bearing capacity of the junctions, and what are the fracture properties of junctions under tensile loading? (iii) What influence does the growth of lateral branches have on the mechanical properties of the junctions?

The knowledge gained from these analyses, together with previously published data on the anatomy and morphology of the junctions and on the biomechanical properties of the tissues involved (Mylo et al., 2021a) will allow for a better understanding of the fracture-prone and fracture-resistant branch-branch junctions of Opuntioideae and plants in general.

## Materials and methods

### Biomechanical analyses

#### Plant material

**FIGURE 1:**
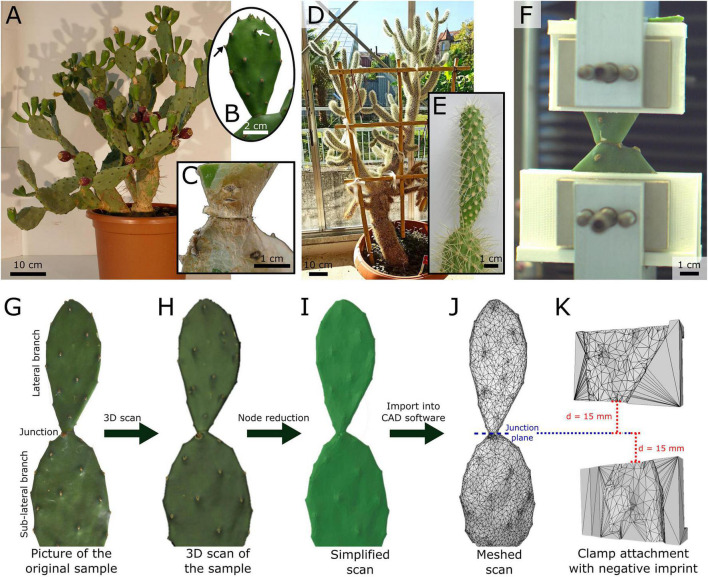
**(A–C)** Exemplary images and representations of the experimental plants and samples of *Opuntia ficus-indica* and **(D,E)** *Cylindropuntia bigelovii*, **(F)** a front view of a junction tensile test including printed clamp attachments, and **(G–K)** the construction process of individualized clamp attachments for optimized form fit during the tensile tests on the junctions of *O. ficus-indica*. **(B)** Sample of *O. ficus-indica* classified as “young” because of as-yet unshed leaves (exemplarily marked by arrows). **(C)** Older lateral junction of *O. ficus-indica* with periderm coverage (brown tissue). **(E)** Lateral junction and branch of *C. bigelovii*. **(G)** Image of an *O. ficus-indica* sample showing the lateral and sub-lateral branches (cropped at the bottom) and the connecting junction. **(H)** Merged 3D scan from multiple individual scans, including surface color representation. **(I)** Simplified model of the scan, reduced to 10,000 nodes and without original surface color. **(J)** Mesh representation of the model, with the position of the junction plane marked as a reference for the alignment of the clamp attachments. **(K)** Mesh representation of one each of the upper and lower clamp attachments with a negative imprint of the *O. ficus-indica* branch mesh. The clamp attachments were positioned with their adjacent edges parallel to the junction plane and at a distance of 15 mm from it. The models served as templates for 3D-printed individualized clamp attachments.

We purchased all the experimental plants from Kakteenland Steinfeld (Steinfeld, Germany) in July 2019. Four plants of *Opuntia ficus-indica* (L.) MILL. (hereafter *O. ficus-indica*) (Figure 1A) were cultivated under controlled conditions in a phytochamber (for detailed conditions refer to Mylo et al., 2020) (young samples were removed after 3 months and old samples after 9 months). One plant of *Cylindropuntia bigelovii* (ENGELM.), F.M. KNUTH (hereafter *C. bigelovii*) (Figure 1D) was cultivated in a greenhouse (natural day–night rhythm, temperature: 18.8 ± 8.5°C, relative humidity 52.3 ± 13.1%; both mean values ± standard deviation) at the Botanic Garden of the University of Freiburg (Germany) (the samples were removed after 15 months). For lateral branches of *O. ficus-indica*, a distinction was made between branches with small leaves still attached (“young lateral branches,” Figure 1B) and those with all leaves shed (“older lateral branches” with periderm formation at the junction and, for some samples, partly on the branch surface, Figure 1C). Fourteen samples each of the young and older junctions were tested. The samples of each of the two groups came from two plants (seven samples from each plant). We tested all healthy available lateral branches of one *C. bigelovii* plant (fifteen samples), and all had shed their leaves (Figure 1E). Preliminary tests showed the difficulty involved in removing young *C. bigelovii* samples (with leaves) without failure of the junction making mechanical testing of the junctions impossible. All the experimental plants were well-watered 3 days before the mechanical tests.

#### Biomechanical tensile test

The samples, consisting of the sub-lateral and lateral branches and the connecting junction (Figure 1G), were carefully removed from the experimental plant. If necessary, we trimmed the samples to a length of 60 mm from the junction in both directions in order to fit the size of the clamping device. If this was necessary for the lateral branch, the cut part of the sample was weighed after the sample had been cut (Kern 440–45N; Kern & Sohn GmbH, Balingen-Frommern, Germany; reproducibility: 0.1 g). All cut surfaces were sealed with Parafilm^®^ (Bemis, Neenah, WI, United States), and the spines of parts to be tested were carefully removed using a nail clipper.

At a maximum of 20 min after removal from the plant, the junctions were tested to failure under monotonic uniaxial tensile loading using a universal testing machine (Inspekt mini; Hegewald & Peschke Meß- und Prüftechnik GmbH, Nossen, Germany) equipped with a 1 kN load cell for the tests on *O. ficus-indica* and a 100 N load cell for the tests on *C. bigelovii* (both KAP-S; A.S.T. Angewandte System Technik GmbH, Wolnzach, Germany). The initial clamp distance was set to 30 mm. For the samples with older branches of *O. ficus-indica*, this was not always possible because of the more complex geometry and the limited space in the clamping device, in which case the initial distance was measured using a digital caliper (Mitutoyo Abso-141 lute Digimatic; measuring accuracy: ±0.03 mm; Kawasaki, Japan). The samples were clamped so that the junction was positioned halfway between the upper and lower clamps (Figure 1F). The tensile speed was set to 1 mm/s, with force and displacement being recorded at 50 Hz.

After failure of the sample, the tested part of the lateral branch was weighed, and if it was cut beforehand, the total mass was calculated from the sum of the two parts. To quantify the junction area, the fractured junctions of the lateral and sub-lateral branches, together with a reference scale, were photographed (Lumix DMC-FZ1000 camera; Panasonic Corporation, Kadoma, Japan) and measured by hand using the Fiji software (ImageJ version 1.53c; Schindelin et al., 2012). In some samples, the fractured junction of the sub-lateral branch had been torn out to such an extent that the fracture area could not be measured precisely, resulting in exclusion of this measurement. In all the other samples, the mean of the areas from the lateral and sub-lateral junction parts was calculated and used for further calculations.

#### Individualized clamp attachments

To minimize slipping effects under tensile load, we designed and printed customized clamp attachments with optimal form-fit for each sample of *O. ficus-indica*. For this purpose, the selected lateral and sub-lateral branches (Figure 1G) were 3D scanned (Artec Spider; Algona GmbH, Stuttgart, Germany) while still being attached to the plant. In order to obtain a model that was as accurate as possible, several individual scans were merged in the scanning software (“Autopilot mode,” Artec Studio 12 Professional, version 12.1.6.16: Algona GmbH, Stuttgart, Germany), and any possible holes in the geometry were filled (Figure 1H). The individual models were simplified to 10,000 nodes in order to obtain a manageable data size (Figure 1I), saved as an STL file, and imported as a mesh into the SolidWorks CAD software for further editing (Figure 1J; version 2019, Dassault Systèmes SolidWorks Corporation, Waltham, MA, United States).

We defined a junction plane in the model to allow for a parallel arrangement of the clamps and perpendicular force application to the junction. The ends of the two pairs of the blank attachment clamps (with a base geometry of 40 mm height, 12 mm depth, and up to 80 mm width depending on the size of the sample) were placed at a distance of 15 mm apically and basally to this plane in order to keep the initial clamp distance constant at 30 mm (Figures 1J,H). The scanned branch geometry was extracted from the clamp attachments to obtain a negative imprint (Figure 1K). The resulting four CAD clamp attachment models were 3D-printed using an extrusion printer (Pro2Plus; Raise 3D Technologies, Inc., Irvine, CA, United States) with a polylactic acid filament (Premium PLA; FormFutura BV, Nijmegen, Netherlands) and positioned to fit between the original clamps and the samples during tensile loading (Figure 1F) (refer to Mylo et al. (2020) for the fabrication of equivalent attachments for bending tests).

The 3D scans had to be carried out on branches that were attached to the plant, but this was not possible for *C. bigelovii* because of its growth habit and large number of spines. However, in order to improve the form-fit compared with the standard clamps, clamp attachments with a semi-circular discharge of 15 to 45 mm in diameter were manufactured using the SolidWorks software and the above-mentioned printing device. Once the test branches had been cut and the spines had been removed, the clamp attachment with the most suitable form was selected. Because the growth form and/or the angle between the branches of some samples of *C. bigelovii* did not allow for the use of the upper or lower clamp attachment pair, we used standard clamps (flat with a small regular texture on the surface) for the samples.

#### Biomechanical variables and fracture analysis

After sample failure, we evaluated the failure site with respect to its location (at the junction, through the branch, or with both involved) and the characteristics of the failure surface (smooth, rough, or as a cup and cone failure in which parts of the basal branch were pulled out into a cone shape).

Further processing of the raw force-displacement data was performed using the GNU R software (v.4.0.4; R Core Team, 2019). In order to remove possible preloads caused by sample fixation, the recorded force data were tared over the mean value of the last eight values after the failure of the sample. The values of strain and stress were calculated based on the initial clamp distance and the junction area *A_j_* [m^2^], respectively. We determined the mass of the apical branch *m*_*a_branch*_ [kg] and calculated the area density ρ_*A*_ [kg/m^2^] as a ratio of the mass of the apical branch and the junction area.

**FIGURE 2:**
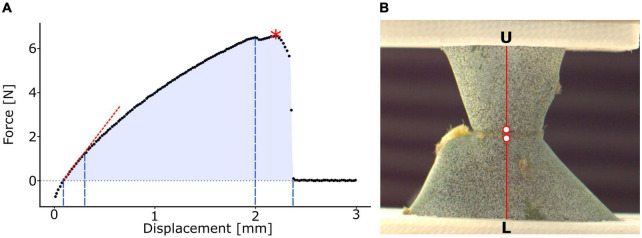
Analysis of tensile test and local surface strains. **(A)** Exemplary illustration of a force-displacement curve. The slope of the first linear intercept as a measure for the tensile stiffness *k* (red dotted line), the point of maximum force *F*_*max*_ (red asterisk), and work of fracture *W* (light blue area) are indicated. The division into the elastic range (first to second dashed blue line), viscoelastic plastic range (second to third dashed blue line), and fracture range (third to fourth dashed blue lines) and their respective displacement ranges are marked. **(B)** Clamped junction of a young sample of *Opuntia ficus-indica* sprayed with a stochastic speckle pattern in an unloaded state. The stochastic speckles improve the surface pattern detection in digital image correlation analysis, thereby enhancing the precision of local strain quantification. The section for detailed strain analysis is marked in red and extends from the lower clamps (L) to the upper clamps (U). The two points, directly basal and apical to the junction, used for junction strain ε_*j*_ analysis are highlighted as red dots with a white filling.

The elastic behavior includes tensile stiffness *k* [N/m] as the slope of the force-displacement curve and effective tensile modulus $\bar{E}$ [N/m^2^ = Pa] as the slope of the stress-strain curve. Both the tensile stiffness and the effective tensile modulus were calculated as the slope of the first linear section (manually selected not considering initial negative force value; c.f. red dotted line in Figure 2A). In this context, “effective” describes the tensile modulus of the entire sample, i.e., the “effective” tensile modulus is a function of the tensile moduli of individual tissues involved, their respective volume fraction, and their three-dimensional arrangement. Sometimes, the term “structural” is used instead of “effective” (Rowe and Speck, 1998).

The fracture behavior, however, includes maximum force *F*_*max*_ [N] (refer to the red asterisk in Figure 2A), tensile strength σ_*max*_ [N/m^2^ = Pa] as maximum stress, strain at *F*_*max*_ε_*fmax*_ [%] as the strain value at maximum stress, work of fracture *W* [Nm] as the discrete integral under the force-displacement curve calculated using the trapezoidal rule (the entire blue area in Figure 2A), and fracture energy *G_f_* [N/m] as the work of fracture relative to the junction area.

To study the elastic and viscoelastic plastic behaviors of the samples under tensile load and at failure, we divided the stress-strain curves (measured from the first crossing of the 0 MPa level in the case of negative pre-stresses to the first value at about 0 MPa after failure) into three sections. The first linear part describes the elastic behavior of the sample. The section from the end of the linear part to the first (pre-)failure of the sample describes the viscoelastic plastic behavior. For better readability, we will refer to this section as “plastic” in the following text despite being aware that viscoelastic components also contribute to the behavior of the curve. The range from this failure to the end of the curve describes the fracture behavior. The relative proportion of the total curve was calculated for each of the three sections.

#### Local surface strain analysis

Over the past few decades, digital image correlation (DIC) has become an integral part of materials science as an analysis technique for noncontact quantification of full-field surface strains (Pan, 2018). In recent years, it has also found an increasing application for the analysis of plant systems with regard to their motion (Sachse et al., 2020) and fracture behavior (Mylo et al., 2022) and for comparisons between plant and artificial material systems (Correa et al., 2020). A one-camera setup is sufficient for in-plane measurements of flat surfaces (2D-DIC) (Pan et al., 2009), whereas a two-camera setup is necessary if samples move out of the image plane or have strongly curved surfaces (3D-DIC) (Sutton et al., 2008). However, the latter method is only applicable if all points of the surface to be analyzed are permanently visible in both cameras. This was not feasible for the samples from *C. bigelovii* because of the pronounced undulation of their branch surface. In order to be able to analyze the local strains of the entire test range, we therefore accepted the slightly less accurate measurements and applied 2D-DIC to all the three groups.

A black stochastic pattern was sprayed onto selected samples of each group (Professional spray paint; Liquitex, Cincinnati, OH, United States) after careful application of an anti-glare spray (Helling 3D Laser Scanning Spray; Helling GmbH, Heidgraben, Germany) to increase surface contrast (Figure 2B). Frontal images of the sample surface were captured during the tensile test at 90 frames per second for *O. ficus-indica* samples using a Basler ace camera (acA2040; Basler AG, Ahrensburg, Germany) equipped with a 35-mm lens (CCTV LM35HC; Kôwa, Nagoya, Japan) and at 50 frames per second for samples of *C. bigelovii* using a MotionPro camera (Y4, Redlake Inc., Tucson, AZ, United States) equipped with a 50-mm lens (Zeiss, Jena, Germany). Image stacks were loaded into GOM Correlate (Professional 2016; GOM GmbH, Braunschweig, Germany) and the first image (without tensile loading) was selected as the reference stage. For surface detection, a facet size of 14 pixels was selected, and the maximum point distance was set to 8 pixels. Major technical strain, as strain in the direction of the largest deformation, was calculated for the surface between the clamps. A plane orthogonal to the junction was selected for detailed analysis of the branch strains (Figure 2B). Because of the large strains occurring directly at the junction, local surface pattern recognition was lost during the tensile test. Therefore, two analysis points were defined (in the DIC software) directly basal and apical to the junction. The strain between the two points was defined as junction strain ε_*j*_ [%].

#### Statistics

Because not all the groups are normally distributed, values are reported using the median value and the respective interquartile range (IQR) for better comparability. Statistical analyses and plotting of the data were performed using the GNU R software (version 4.1.2), including the packages “car” (Fox and Weisberg, 2019), “ggplot2” (Wickham, 2016), “stats,” and “ggpubr.”

The analyzed mechanical variables for each plant (two each of *O. ficus-indica* with young and older lateral branches and one of *C. bigelovii*) were tested for normal distribution (Shapiro-Wilk test, α = 0.05) and the variance homogeneity between them (Levene’s test, α = 0.05). To test for differences between plants, we conducted either a one-way analysis of variance (ANOVA) with Tukey’s HSD *post hoc* test (given a normal distribution for all plants and variance homogeneity between plants) or a Kruskal-Wallis test with a Holm-corrected Dunn *post hoc* test (given if at least one of the two assumptions was not met). As no significant difference between the two individual plants of *O. ficus-indica* could be found for any of the analyzed variables (α = 0.05), the data were pooled. Subsequently, the normal distribution, homogeneity of variance, and differences between the groups (junctions with young and older lateral branches of *O. ficus-indica* and junctions of *C. bigelovii*) were tested again according to the described procedure. Levels of significance were as follows: *p* ≥ 0.05: not significant (n.s.); 0.05 > *p* ≥ 0.01: significant (*); 0.01 > *p* ≥ 0.001: very significant (^**^); 0.001 > *p*: highly significant (^***^).

To test whether the mechanical variables were related to the mass of the lateral branch, we performed a (linear) correlation analysis using Pearson’s correlation coefficient (Pearson’s ρ). The direction (positive or negative) of the correlation is indicated by the sign of Pearson’s ρ, and its absolute value classifies the strength of the correlation, with |ρ| < 0.3 indicating no correlation, 0.3 ≤ |ρ| < 0.5 indicating a weak correlation, 0.5 ≤ |ρ| < 0.7 indicating a medium correlation, and 0.7 ≤ |ρ| indicating a strong correlation (adapted from Kampowski et al., 2017; Mylo et al., 2021b).

### Finite element analysis

We analyzed the elastic behavior of cacti junction models using continuum elasticity. All the simulations reported here used the small strain formulation of elasticity with a linear isotropic elasticity model. Young’s modulus and Poisson’s ratio were varied spatially to reflect to the heterogeneous composite characters of the branches and the junction. For better readability, we will summarize the epidermis and hypodermis, and in the older branches of *O. ficus-indica* the periderm, as dermal tissues, being aware that the hypodermis is not a part of dermal tissues in origin although it functions in unison with the epidermis. We distinguished the mechanical properties of dermal tissues, vascular bundles, and the parenchyma. The mechanical properties of both dermal tissues and vascular bundles and respective geometries from magnetic resonance imaging scans (MRI) and 3D scans were obtained in independent experiments (Mylo et al., 2021a). We distilled a simplified geometric model of the junction from the measurements (for more details refer to Supplementary Table 1).

#### Creation of geometric models

Based on the MRI scans of the *O. ficus-indica* junctions, a “standardized geometric model” was created, in which centrosymmetrically arranged tissue layers were simplified as shells. From the outside to the inside, the geometric model consisted of an outer shell of dermal tissues, a chlorenchyma shell, a vascular bundles shell, and a central parenchyma core. The thickness of the shell of dermal tissues was set at a constant 0.22 mm according to Mylo et al. (2021a). In order to calculate the shell thickness and location of the vascular bundles, the total area and the summed area of the vascular bundles of a photographed branch cross-section and a junction of *O. ficus-indica* sample with a young lateral branch were manually measured using the Fiji software.

Young’s modulus of the dermal tissues was set at 40 MPa for simulations of *O. ficus-indica* with young lateral branches (“Opuntia_model_young”) and *C. bigelovii* (“Cylindropuntia_model”). Because of the periderm formation around the junction of older lateral branches of *O. ficus-indica*, Young’s modulus of the dermal tissues including the periderm was set at 440 MPa (“Opuntia_model_older”). For vascular bundles, Young’s modulus was set at 1,200 MPa for both Opuntia_models and at 7.5 MPa for the “Cylindropuntia_model” (Mylo et al., 2021a). The elastic modulus of the chlorenchyma and parenchyma could not be independently determined experimentally. Thus, we used a value of 0.24 MPa representing the mean value of the parenchyma from succulent plant leaves (*Delosperma ecklonis* and *Delosperma cooperi*; Hesse et al., 2020) for the chlorenchyma shell and the parenchyma core in all our models. We were aware that the overall behavior of the parenchyma was likely to be governed by poroelasticity, but we were only interested in the approximate elastic behavior of the branch junction and only aimed to obtain a simplified description of the mechanical properties. Additionally, a quantitative model of the poroelastic behavior of the junction would have required a large set of time-dependent measurements. Although we carried out additional calculations using the finite strain formulation of elasticity, the results of these calculations did not yield any insights beyond the small strain results reported here.

Following standards practices, we discretized the governing (elastostatic) equations using the finite element method (FEM). All the simulations employed Lagrange elements. The geometric model was constructed in SALOME, saved as a boundary representation (BREP) file, and gridded using gmsh. The resulting MSH file was converted into an xdmf/h5 file using meshio and imported into DolphinX (version 0.1.0).

#### Tensile tests

DolphinX was used as the numerical solver by applying Dirichlet boundary conditions on the top and bottom surfaces of the created geometric model. The bottom surface was fully clamped, and for the top surface, only displacement in the direction of tension was allowed. An arbitrary displacement of 0.01 mm was chosen for the Dirichlet condition on the top surface. The corresponding reaction force was calculated with the virtual work principle and by elastic energy.

## Results

The analysis of the tensile tests on the branch-branch junctions included their elastic behavior, which we additionally evaluated by FEM simulation, and the fracture behavior with respect to fracture modes, morphometric and biomechanical variables, and local strains.

### Analyses of elastic behavior

#### Biomechanical analysis in the linear elastic range

**FIGURE 3:**
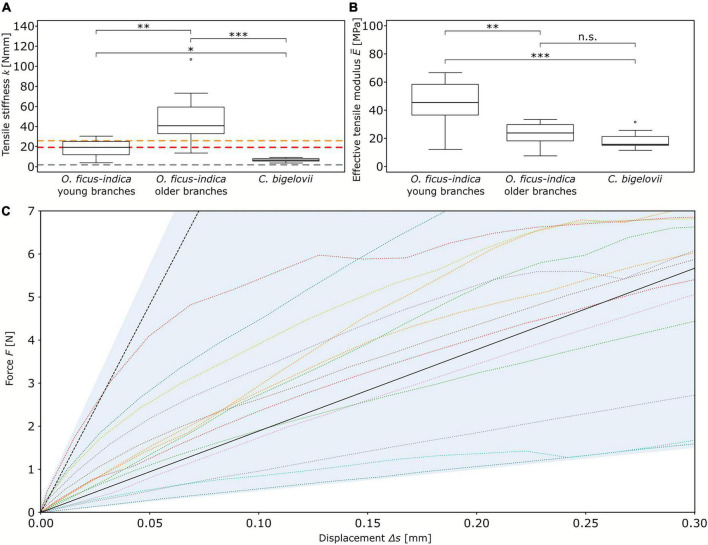
Elastic behavior of the branch-branch junctions under tensile loading. Box plots of the **(A)** tensile stiffness *k* and **(B)** effective tensile modulus $\bar{E}$ of the three analyzed groups. The colored dashed lines in **(A)** mark the tensile stiffness values obtained from “Opuntia_model_young” (red line), “Opuntia_model_older” (orange line), and “Cylindropuntia_model” (gray line). The sample size was 14 for the two groups of *O. ficus-indica* and 15 for *C. bigelovii*. Levels of significance between the groups are marked as follows: *p* ≥ 0.05: not significant (n.s.); 0.05 > *p* ≥ 0.01: significant (*); 0.01 > *p* ≥ 0.001: very significant (^**^); 0.001 > *p*: highly significant (^***^). **(C)** Force-displacement curves of the junctions of *O. ficus-indica* with young lateral branches tested under tensile loading (colored dotted lines, with the range of the data shaded in light blue) and the simulation of the FE models (black lines). The dotted black line refers to the “standardized geometric model” in which the thickness of the shell of vascular bundles corresponds to the biological value, whereas the solid black line refers to the “adjusted geometric model” with a reduction in thickness of the shell of vascular bundles making up for their net-like structure.

The tensile stiffness *k* differed significantly among the three groups, with the highest value for older lateral junctions of *O. ficus-indica* (median: 40.4 N/mm), an intermediate value for young lateral junctions of *O. ficus-indica* (median: 19 N/mm), and the lowest value for *C. bigelovii* (median: 6.3 N/mm) (Figure 3A). For the effective tensile modulus, no significant differences were found between older lateral junctions of *O. ficus-indica* (median: 23.7 MPa) and junctions of *C. bigelovii* (median: 15.8 MPa), with values of both groups being significantly lower than those of the young lateral branches of *O. ficus-indica* (median: 45.5 MPa) (Figure 3B). Values of the individual samples can be found in Supplementary Table 2 and a detailed statistical analysis in Supplementary Table 3.

For the tensile stiffness *k*, the positive linear correlation with the apical branch mass *m*_*a*_*branch*_was strong for *O. ficus-indica* (young branches) and medium for *O. ficus-indica* (older branches) and *C. bigelovii.* The effective tensile modulus $\bar{E}$showed a weak positive linear correlation for *O. ficus-indica* (young branches), no correlation for *O. ficus-india* (older branches), and a medium negative correlation for *C. bigelovii* (Table 1).

**TABLE 1:** Correlation results of the elastic behavior variables under tensile loading with the lateral branch mass for all the three analyzed groups.

	Correlation with apical branch mass *m_a_branch_*		
	***O. ficus-indica***	***O. ficus-indica***	***C. bigelovii***
	Young lateral branch	Older lateral branch	
	
	*N* = 14	*N* = 14	*N* = 15

	**Tensile stiffness** **k**		
Pearson‘s ρ	0.819	0.588	0.543
	**Effective tensile modulus** $\bar{E}$		
Pearson‘s ρ	0.460	−0.204	−0.512

The coefficients for linear correlation (Pearson’s ρ) and sample size (*N*) are presented.

#### Finite element models

**FIGURE 4:**
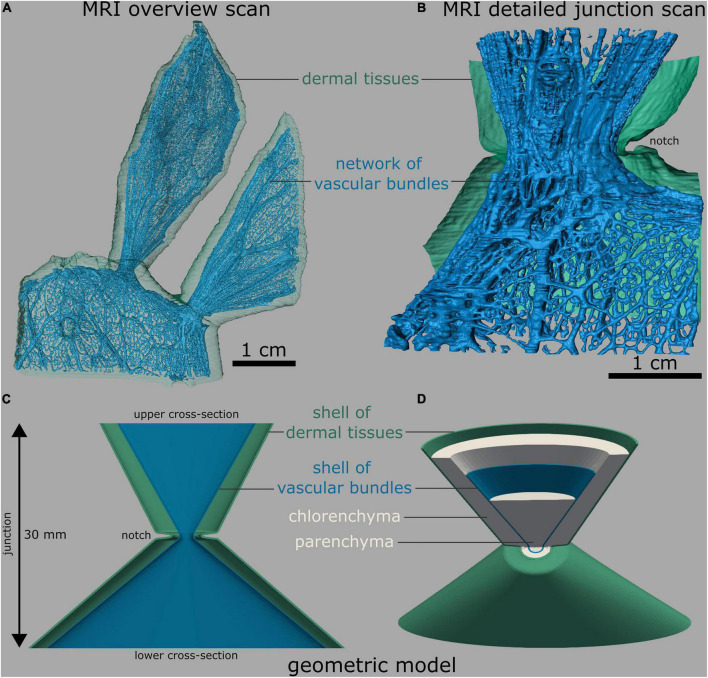
Branch-branch junctions of *O. ficus-indica*. **(A,B)** Segmented magnetic resonance imaging scans (adapted from Mylo et al., 2021a). **(C,D)** Finite element mesh. **(A)** Overview scan with one sublateral branch and two connected lateral branches. The net-like arrangement of the vascular bundles is visible. **(B)** Detailed image of a branch-branch junction with a bisected layer of dermal tissues (consisting of the epidermis, hypodermis, and, in older branches of the periderm) for better visibility. **(C)** Bisected front view and **(D)** tilted view with bisected upper half of the “standardized geometric model,” consisting of five shells, namely, an outer shell of dermal tissues (green), a chlorenchyma shell (gray), a vascular bundle shell (blue), and a central parenchyma core (gray). For better illustration, all the shells are shown only in **(D)**.

Based on a quantitative analysis of the morphology and anatomy of the biological samples (Figures 4A,B), we constructed an FE “standardized geometric model” of the junction with a total height of 30 mm (Figures 4C,D, cf. Supplementary Table 1). Its upper cross-section was oval with radii of 11.5 and 4.5 mm corresponding to an area of 162.6 mm^2^. The radii of the lower cross-section were 21 and 4.5 mm, which corresponded to an area of 296.9 mm^2^. The inner shell (vascular bundles, colored blue in Figure 4), likewise oval, had radii of 9.1 and 2.1 mm at the top and 18.6 and 2.1 mm at the bottom. The initial shell thickness of the vascular bundles was 0.13 mm at both the top and bottom with a linear gradient toward the junction at which it had a thickness of 0.2 mm. With respect to the values of the biological samples, we created an “adjusted geometric model” in which all geometric and mechanical properties were kept constant with the exception of the cross-sectional area of the vascular bundles. To account for the simplification of the net-like structure of the vascular bundles as a shell, we reduced the cross-sectional area of the shell by 81.9%, resulting in a shell thickness of 0.024 mm (top and bottom) and 0.039 mm at the junction. The outer radius of the circular junction was 2 mm, and that of the shell of vascular bundles was 1 mm (Figures 4C,D). We created three cactus models from the “adjusted geometric model.”

#### Comparison of experimental and simulated data

The tensile stiffness of the “standardized geometric model” ranged toward the upper end of the values of the *O. ficus-indica* samples with young lateral branches (dashed black line compared with colored lines in Figure 3C). In contrast, the “adjusted geometric model” (equal to “Opuntia_model_young”) gave a tensile stiffness of 18.9 N/mm (solid black line in Figure 3C and red dashed line in Figure 3A), which deviated by about 0.5% from the median value of the plant data (19 N/mm). The tensile stiffness of “Opuntia_model_older” was 25.5 N/mm (orange dashed line in Figure 3A), which equated to a 36.9% reduction compared with the biological junctions. Furthermore, “Cylindropuntia_model” had a tensile stiffness of 1.7 N/mm (gray dashed line in Figure 3A), which corresponded to a 75.4% reduction compared with that of the plant junctions.

**FIGURE 5:**
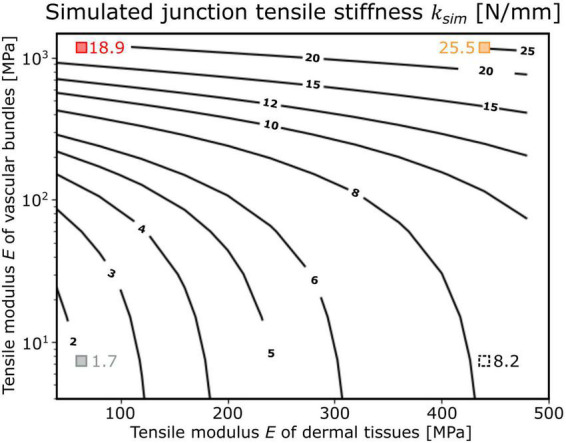
Dependence of the tensile stiffness *k*_*sim*_ of the “adjusted geometric models” (shown as isobar lines with the corresponding values) as a function of the tensile moduli *E* of the shell of dermal tissues and the shell of vascular bundles (both with constant thickness). The simulated junction tensile stiffness of the respective models is marked as red square that corresponds to “Opuntia_model_young,” orange square that corresponds to “Opuntia_model_older,” gray square that corresponds to “Cylindropuntia_model,” and black dashed square that corresponds to “Cylindropuntia_model” with a stiffened shell of dermal tissues and was only applied as a theoretical comparison.

By changing the tensile modulus of the shells of the dermal tissues and the range found for the investigated biological tissue properties, the influence of the dermal tissues on the overall tensile stiffness was increased with decrease in tensile modulus of the vascular bundles (Figure 5). For a tensile modulus of 1,200 MPa of the shell of vascular bundles, an increase in the tensile modulus of the shell of dermal tissues from 40 to 440 MPa led to an increase in overall stiffness of about 34.9% (from 18.9 to 25.5 N/mm; red and orange square in Figure 5),whereas for a tensile modulus of the vascular bundles of 7.5 MPa, the same change in tensile stiffness of the shell of dermal tissues resulted in an increase of about 382.4% (from 1.7 to 8.2 N/mm; gray and black dashed square in Figure 5) in the tensile stiffness of the overall system.

### Fracture analyses

#### Fracture modes

**FIGURE 6:**
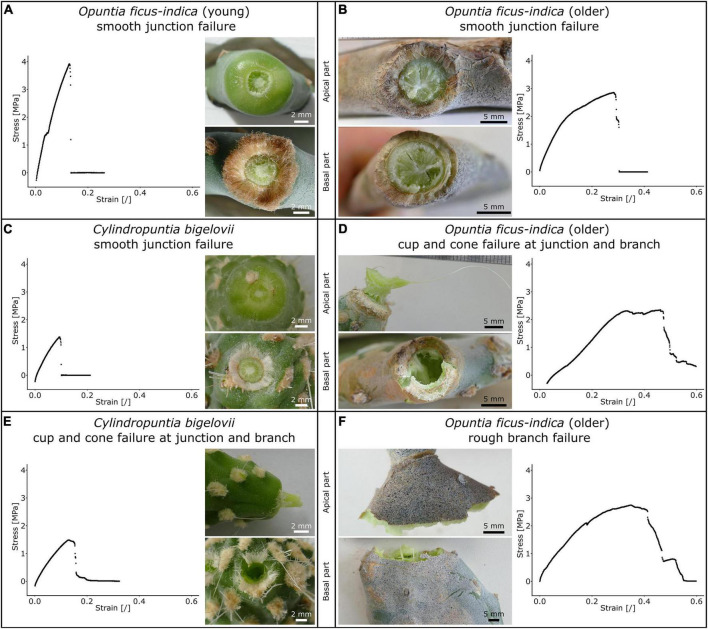
Stress-strain curves of tensile tests until failure (for better comparability, the diagram axes have identical ranges) and images of the apical and basal fracture sites of the lateral junctions of *Opuntia ficus-indica* and *Cylindropuntia bigelovii.* Smooth junction failure of **(A)**, a young lateral *O. ficus-indica* junction with a large number of trichomes around the junction, **(B)** an older lateral *O. ficus-indica* junction with ruptures of the periderm coverage (brown tissue covering the epidermis), and **(C)** a junction of *C. bigelovii*. Cup and cone failure of **(D)** an older lateral *O. ficus-indica* junction (with strands of vascular bundles having been pulled out in the apical junction part and ruptures in the basal junction part) and of **(E)** a junction of *C. bigelovii* (consisting of the parenchyma and strands of vascular bundles) with failure at the junction and basal branch. **(F)** Rough branch failure of an older lateral *O. ficus-indica* junction with protruding strands of vascular bundles. The junction shows signs of elongation, but no pre-failure effects can be detected. We sprayed a stochastic pattern onto the surface of the samples shown in **(B,F)** to improve facet detection for digital image correlation analysis.

Table 2 shows the distribution of tensile failure with respect to fracture site and fracture surface under tensile load (refer also to Supplementary Table 2). In total, three fracture modes occurred with various percentage distributions in the samples. (i) Tensile failure directly at the junction with a smooth fracture surface (Figures 6A–C) was most commonly found in the young *O. ficus-indica* samples. In the older samples of *O. ficus-indica*, the failure was also classified as smooth when some parenchyma tissues but no vascular bundles protruded (Figure 6B). (ii) In the case of cup and cone failure (Figures 6D,E), which was extremely common in *C. bigelovii*, the cone that was torn out of the adjacent basal or apical branch always consisted of vascular bundles embedded in the parenchyma. The older junctions of *O. ficus-indica* exhibited cup and cone failure (Figure 6D) with visible ruptures to the periderm covering around the junctions. (iii) Failure in the branch with a rough fracture surface was only observed in the older *O. ficus-indica* samples, with the failure running (at least partially) along the clamps and, in two samples, exclusively through the branch (Figure 6F). A visual scatter plot analysis of the analyzed mechanical variables showed no clustering of failure site (Supplementary Figure 1), and so the data were pooled.

**TABLE 2:** Fracture modes of the investigated samples, with percentages in bold indicating the most common fracture mode under tensile loading (highest values in bold).

Sample	Fracture site	Fracture surface	Sample percentage	Sample size *N*
*Opuntia ficus-indica* young	junction	smooth	**93%**	14
	junction and branch	cup and cone	7%	
*Opuntia ficus-indica* older	branch	rough	**64%**	14
	junction	smooth	22%	
	junction and branch	cup and cone	14%	
*Cylindropuntia bigelovii*	junction	smooth	20%	15
	junction and branch	cup and cone	**80%**	

Table 3 presents the relative strain ranges of the elastic, plastic, and fracture behaviors (details are given in Supplementary Table 2) with respect to total strain. The total strain of older *O. ficus-indica* samples was approximately twice as high as that of the other samples. The elastic range had the lowest relative proportion in all the three groups. In the young junctions of *O. ficus-indica* and those of *C. bigelovii*, the plastic range had the largest relative proportion of the total strain. Median fracture and plastic range of about the same size in the older junctions of *O. ficus-indica*, with the fracture range being slightly larger.

**TABLE 3:** Percentage portion of the elastic, viscoelastic-plastic, and fracture behaviors of total strain under tensile loading (highest values in bold).

Sample	Total strain [/]	Elastic range [%]	Viscoelastic plastic range [%]	Fracture range [%]	Sample size *N*
*Opuntia ficus-indica* young	0.25 (0.06)	20.2 (6.4)	**74.5** (12.1)	4.4 (2.3)	14
*Opuntia ficus-indica* older	**0.48** (0.22)	11.7 (21.1)	44.8 (27.8)	**47.2** (14.4)	14
*Cylindropuntia bigelovii*	0.18 (0.05)	17.1 (9.4)	**49.0** (19.7)	27.5 (17.9)	15

The median and interquartile ranges (in brackets) are given for each, together with the sample size *N* for each group.

#### Biomechanical analysis at failure

**FIGURE 7:**
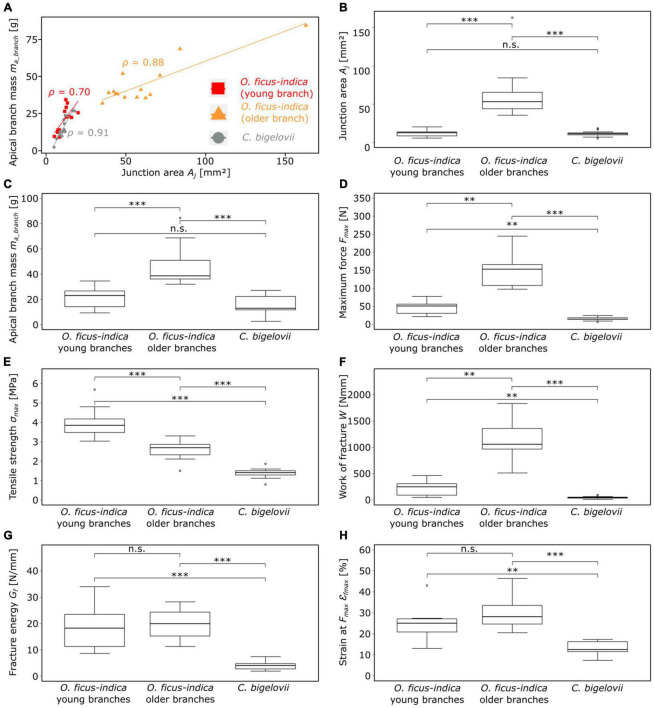
Plots and statistical comparisons of various geometric, physical, and biomechanical variables of the lateral branch-branch junctions of *Opuntia ficus-indica* (with distinction between samples with young and older lateral branches) and *Cylindropuntia bigelovii*. **(A)** Scatter plot of the apical branch mass against the junction area. The linear regression line and the corresponding Pearson’s ρ-value are indicated for each of the three groups (red = young lateral *O. ficus-indica* branches, orange = older lateral *O. ficus-indica* branches, gray = *C. bigelovii*). Box plots of the **(B)** junction area, **(C)** apical branch mass, and **(D–H)** several fracture variables calculated from the force-displacement and stress-strain curves of the tensile tests until failure are presented. The sample size for all the presented variables was 14 for both groups of *O. ficus-indica* and 15 for *C. bigelovii*. Levels of significance between the groups are marked as follows: *p* ≥ 0.05: not significant (n.s.); 0.01 > *p* ≥ 0.001: very significant (^**^); 0.001 > *p*: highly significant (^***^).

Preliminary tests showed that the young lateral branches of *O. ficus-indica* also fell off under rather low external forces, and so we made a distinction between junctions with young and with older lateral cladodes for this species. When we prepared the samples of *C. bigelovii*, three (even at the stage of shed leaves) experienced branch break-off before completion of the test. All the samples among the five lightest samples, reflected the enormously high fragility of particularly the youngest branches. A comparison of the junction area and mass of the lateral branches (Figures 7A–C) indicates that the young samples of *O. ficus-indica* with their leaves still attached are comparable in size and mass to the lateral samples of *C. bigelovii* (area density > 1 g/mm^2^). In addition, the distinction between the younger and older lateral branches of *O. ficus-indica* can be made based on the presence or absence of leaves.

A strong correlation (Pearson’s ρ ≥ 0.7) between the mass of the lateral branch and the area of the junction was found for all the three groups (Figure 7A). No significant differences between the samples of *O. ficus-indica* with young lateral branches and those of *C. bigelovii* were found for the junction area and the apical branch mass. However, both groups differed significantly from the samples of *O. ficus-indica* with older lateral branches for the two variables (Figures 7B,C). The values of the junction area ranged from 5.1 mm^2^ (*C. bigelovii*) to 162.9 mm^2^ (*O. ficus-indica* with older branches) and those of the lateral branch mass from 2.5 g (*C. bigelovii*) to 84.4 g (*O. ficus-indica* with older branches). The median values of area density were >1 for the young junctions of *O. ficus-indica* (1.75 g/mm^2^) and *C. bigelovii* (1.26 g/mm^2^) and <1 for the older junctions of *O. ficus-indica* (0.77 g/mm^2^), with significant differences among all the three groups (all *p*-values < 0.01).

With regard to maximum force, all the three groups differed significantly from each other, with *C. bigelovii* having the lowest median value (14.4 N), *O. ficus-indica* with young lateral branches having the intermediate median (51.2 N), and *O. ficus-indica* with older lateral branches having the highest median (153 N) (Figure 7D). All the three groups also differed significantly from each other in tensile strength, with the highest median for *O. ficus-indica* with young lateral branches (3.84 MPa), the intermediate median for *O. ficus-indica* with older lateral branches (2.70 MPa), and the lowest median for *C. bigelovii* (1.41 MPa) (Figure 7E). Likewise, the three groups differed significantly in the work of fracture, with the lowest median for *C. bigelovii* (44.6 Nmm), the intermediate median for *O. ficus-indica* with young lateral branches (250.7 Nmm), and the highest median for *O. ficus-indica* with older lateral branches (1,059.5 Nmm) (Figure 7F). For fracture energy, no significant differences were found between the samples of *O. ficus-indica* with young branches (median: 18.23 N/mm) and older branches (median: 19.91 N/mm). Both groups differed significantly from the samples of *C. bigelovii* (median: 4.05 N/mm) (Figure 7G). Similarly, no significant differences were found for the strain at *F*_*max*_ between samples of *O. ficus-indica* with young (median: 25%) and older (median: 28.2%) lateral branches. Both groups were significantly different from the samples of *C. bigelovii* (median: 12.6%) (Figure 7H). Values of the individual samples can be found in Supplementary Table 2 and a detailed statistical analysis in Supplementary Table 3.

Maximum force showed a strong linear correlation with the lateral branch mass for all the three groups. For tensile strength, *O. ficus-indica* (young branches) showed a medium positive linear correlation, *O. ficus-indica* (older branches) a weak negative correlation, and *C. bigelovii* no correlation. The work of fracture of the young and older branches of *O. ficus-indica* revealed a strong positive linear correlation and in *C. bigelovii* a medium positive linear correlation with lateral branch mass. For fracture energy, *O. ficus-indica* (young branches) showed a strong positive linear correlation, whereas no correlation was found for *O. ficus-indica* (older branches) and *C. bigelovii*. Weak positive linear correlations for strain at *F*_*max*_ were found for *O. ficus-indica* (young branches), and no correlation for the *O. ficus-indica* (older branches) and *C. bigelovii* samples (Table 4).

**TABLE 4:** Correlation results of the fracture tensile variables with the lateral branch mass for all the three analyzed groups.

	Correlation with apical branch mass		
	***O. ficus-indica***	***O. ficus-indica***	***C. bigelovii***
	Young lateral branch	Older lateral branch	
	
	*N* = 14	*N* = 14	*N* = 15

	**Maximum force**		
Pearson‘s ρ	0.907	0.853	0.809
	**Tensile strength**		
Pearson‘s ρ	0.620	−0.386	−0.027
	**Work of fracture**		
Pearson‘s ρ	0.833	0.773	0.571
	**Fracture energy**		
Pearson‘s ρ	0.721	−0.252	0.176
	**Strain at *F*_*max*_**		
Pearson‘s ρ	0.333	−0.105	0.245

The coefficients for linear correlation (Pearson’s *ρ*) and sample sizes (*N*) are presented.

### Local strain analysis

**FIGURE 8:**
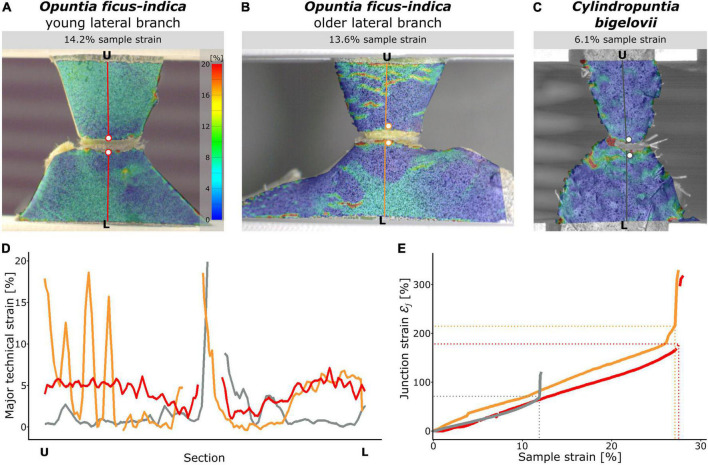
Local strain analysis during tensile loading of the junction samples using the digital image correlation technique. The surface strains (major strain) of selected samples of **(A)** *Opuntia ficus-indica* with a young lateral branch, **(B)** *O. ficus-indica* with an older lateral branch, and **(C)** *Cylindropuntia bigelovii* at the stage of about the respective half maximum sample strain are shown color-coded (scale in **(A)** applies to all the three sub-figures). Please note that large local strains resulted in loss of surface detection at the junctions. Constructed sections running orthogonally from the lower clamping (L) to the upper clamping (U) midway through the junctions are marked in color. The two points directly basal and apical to the junction (colored dots with a white filling) are used to calculate the junction strain. **(D)** Course of the local strains at the given sample strain along the sections (each normalized to the length from L to U). For better comparability, the *Y*-axis is limited to 20% strain; larger strain values occurred only directly at the junction and were calculated as junction strain ε_*j*_. **(E)** Junction strains over the duration of the tensile test represented as sample strain. The dashed horizontal and vertical lines mark the failure of the respective samples. In **(D,E)**, the red curves belong to a sample of *O. ficus-indica* with a young lateral branch, the orange curves to a sample of *O. ficus-indica* with an older lateral branch, and the gray curves to a sample of *C. bigelovii*.

The digital image correlation analysis revealed a local concentration of surface strains during tensile loading in the region at and directly around the junction (Supplementary Videos 1–3). Because of the large deformations and protrusion of the junction, a loss of surface pattern recognition at the junction occurred with increase in sample strain (strain relative to the initial distance between the upper and lower clamps). The maximum local strain before junction failure could therefore not be measured using the section analysis (refer to missing peaks in Figure 8D). However, local strains of the lateral and sublateral branches could be measured with hardly any loss of surface detection during the entire tensile test. For better comparability, the section analysis of the three species was analyzed in detail at about half the maximum sample strain. Surface strain analyses covering the entire tensile tests can be found in Supplementary Videos 1–3. For *C. bigelovii*, only a small deformation of the branches was measured (about 1–1.5% strain), and strain values of about 5% were found only directly basal to the junction (Figures 8C,D). In both samples of *O. ficus-indica*, stronger deformations of up to 7–8% were visible in large parts of the analyzed branch surface. However, directly basal and apical to the junction, the deformations were markedly lower (Figures 8A,B,D), resulting in a V-shaped pattern directly basal to the junctions in which almost no strain occurred. The older *O. ficus-indica* samples showed a stripe pattern in the lateral branch, with strains oscillating between about 1 and 15–20%. Additional local strain peaks were found in the positions of the areoles in all the three groups.

The junction strainε*_j_* was increased almost linearly for all the three groups throughout the tensile test, with a slightly steeper increase for the samples of *O. ficus-indica* with older lateral branches (Figure 8E). Values at sample failure were lowest for *C. bigelovii* at about 71%, intermediate for *O. ficus-indica* samples with young lateral branches at about 178%, and highest for *O. ficus-indica* samples with older lateral branches at about 220%. They were thus all about six to eight times higher than their respective sample strain at failure ε_*f*_. The steep slope of all three curves after sample failure was caused by the snapping back of the two loaded branch parts.

### Compilation of biomechanical and geometric studies

**FIGURE 9:**
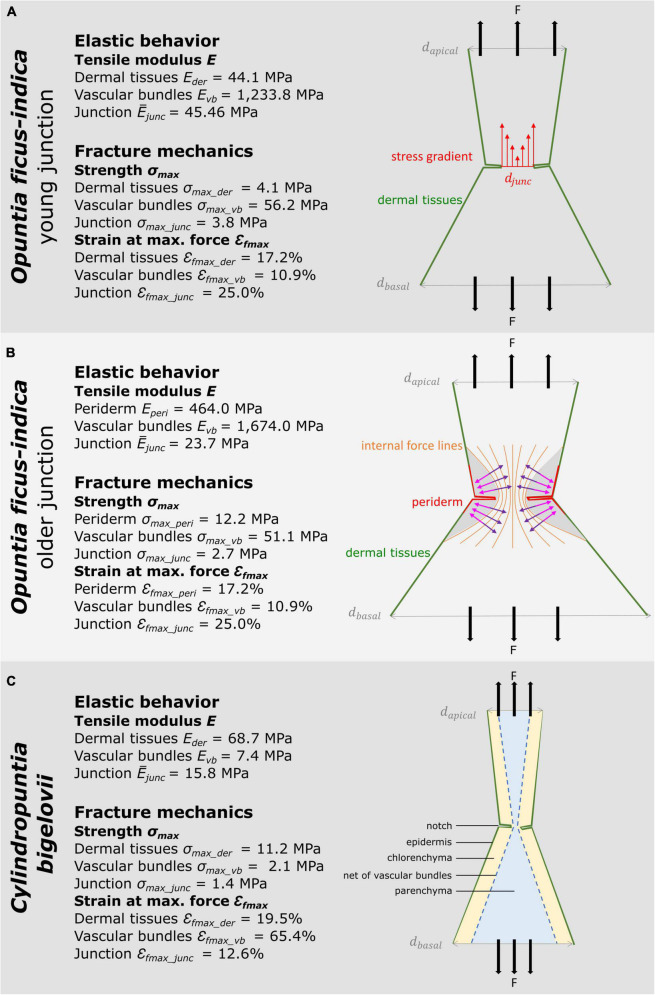
Schematic drawings of the investigated cactus samples with medians of the material properties of tissues (Mylo et al., 2021a) and of entire junction samples derived from the tensile tests. The sub-images illustrate various aspects of the notch effect. **(A)** Young junction of *Opuntia ficus-indica*. Here, we illustrate the stress distribution in the circumferential notch. Since the diameter of the junction *d*_*junc*_ is smaller than the apical *d*_*apical*_ and the basal diameter *d*_*basal*_, a stress gradient (red arrows) results, and the notch stresses reveal peak values. **(B)** Older junction of *O. ficus-indica*. In this sub-image, we visualize the influence of the notches, as intensified by the stiff periderm coating, on the internal force lines. According to their Poisson’s ratio, the junctions show a transverse contraction (purple arrows) with simultaneous longitudinal elongation under tensile loading. However, the gray-marked zones are not stretched in the longitudinal direction by the tensile loading. Instead, they generate a transverse force directed outward (pink arrows) preventing transverse contraction in this area (after “Kerbwirkung” by Ralf Pfeifer, licensed under CC BY-SA 3.0, available at https://commons.wikimedia.org/wiki/File:Kerbwirkung.svg). **(C)** Junction of *Cylindropuntia bigelovii*. This sub-figure focuses on the distribution and structure of the five tissue layers. Under high tensile loading, the dermal tissues can first “unfold” in the area of the notches, and the network of strands of vascular bundles can stretch before the tissues are directly loaded in tension.

The following section compiles the results of the biomechanical and geometric analyses of *O. ficus-indica* and *C. bigelovii* based on the biomechanical and geometric data of the present study and previously published data on the individual tissues (Mylo et al., 2021a). The interplay of the five tissue layers with their characteristic thicknesses and mechanical properties determines the elastic behavior and fracture mechanics of the respective branches and junctions. In Figure 9, we provide the median tensile modulus of the dermal tissues, strands of vascular bundles (Mylo et al., 2021a), and all junctions of *O. ficus-indica* (Figures 9A,B) and *C. bigelovii* (Figure 9C) derived from the tensile tests. The compilation in the Figures should give an indication of the influence of the individual tissues. However, a simple summation of the tensile stiffness of the individual tissues does not match the experimentally obtained values of the junctions. On the one hand, deviations arise because the tissues do not lie individually next to each other but are intergrown in the branch and because the properties of the individual tissues influence each other. Using our “geometric models,” we have been able to show such combined effects on the tensile stiffness of the simulated junctions using the tensile moduli of dermal tissues and vascular bundles as an example (cf. Figure 5). On the other hand, Figures 9A,B reveal that because of the notches at the junctions, not all tissues are tensioned equally. We can assume that the loop-like structure of dermal tissues in the area of the notches is first pulled apart slightly before the dermal tissues as a material comes under tensile load. Furthermore, the notches change the stress distribution in the sample. Figure 9A shows that the notches narrow the diameter in the junction area leading to stress peaks close to the notches. Furthermore, as can be seen in Figure 9B, a transverse contraction under tensile loading is prevented in areas near the notches (marked in gray). The distribution of the five tissue layers depicted in Figure 9C is similar in all the cactus samples and indicates that the net of strands of the vascular bundles accommodates the highest stresses at the notches.

## Discussion

### Elastic behavior

The linear elastic behavior of entire branches, junctions, and single tissues in cacti is one of several xeromorphic adaptations to large fluctuations in water availability in their native (semi-)arid environment. As soon as water is available, their parenchyma can store large amounts of water within a short time leading to rapid swelling of the organs of these succulent plants. In the case of water shortage, however, the organs shrink once again. In particular, the dermal and underlying tissues (e.g., the epidermis with cuticle, hypodermis, and periderm covering) must withstand large volume fluctuations undamaged in order to protect the plant from infestation by invading pathogens. The net-like structures of the vascular bundles can also cope with large volume changes without individual bundles breaking off ensuring undisturbed water transport over long distances in the plant. In addition, wide-band tracheids with annular, helical, or double helical secondary wall thickening are specialized to prevent cell collapse under drought stress and to allow for reversible rehydration once sufficient water supplies are available (Mauseth et al., 1995). In *O. ficus-indica*, wide-band tracheids are embedded in the parenchyma basally and apically of the junction, where vessel elements and fibers predominate. In *C. bigelovii*, wide-band tracheids are only sparsely within the junction where vessels and tracheids predominate (Mylo et al., 2021a).

A comparison of the linear elastic behavior under tensile loading has revealed that the tensile stiffness of the older lateral junctions of *O. ficus-indica* is higher than that of the younger ones by a factor of about two (Figure 3A), and that the effective tensile modulus of the older junctions is almost half that of the younger ones (Figure 3B). Since the tensile modulus of the dermal tissues drastically increases (by periderm accumulation) and that of the vascular bundles moderately increases (Figures 9A,B; Mylo et al., 2021a) with age, we can assume an allometry of the involved tissues during the growth of the junction (Niklas, 1994; Shingleton, 2010; Kaminski et al., 2017). This means that the non-lignified chlorenchyma and parenchyma, with their relatively low tensile modulus (Gibson, 2012; Hesse et al., 2020) and thus small contribution to the effective tensile modulus of the junction, will increase the relative proportion of the cross-sectional area during branch growth. In turn, the cross-sectional proportion of dermal tissues and vascular bundles is decreased in relation to parenchymatous tissues. The accumulated parenchyma allows for cacti to make use of their branches as water-storage organs, which are necessary for survival on the account of environmental conditions in which these xeromorphic plants live (Benson, 1982; Nobel, 2002).

Relative tissue proportions might also be the reason that the median tensile stiffness of *C. bigelovii* junctions is significantly lower than that of the young and older *O. ficus-indica* junctions, although no significant difference in effective tensile modulus has been found with respect to the older *O. ficus-indica* junctions (Figures 3A,B). Compared with the older *Opuntia* junctions, those of *C. bigelovii* have a significantly thinner dermal tissue with an elastic modulus that is one magnitude smaller than that of the dermal tissue with periderm; the vascular bundles of *C. bigelovii* are three-times larger in diameter with an elastic modulus that is three magnitudes smaller than those of the older *Opuntia* junctions (Mylo et al., 2021a). Compared with the young *Opuntia* junctions, those of *C. bigelovii* have a significantly thinner dermal tissue with a comparable elastic modulus; the vascular bundles of *C. bigelovii* are seven times larger in diameter but have an elastic modulus that is three magnitudes smaller than those of the young *Opuntia* junctions (Figure 9C). Thus, a larger relative proportion of dermal tissues and vascular bundles might compensate for the comparable or lower elastic modulus of *C. bigelovii* compared with the same tissues of *O. ficus-indica*.

Comparable conclusions can also be drawn from finite element simulations of the geometric models. The “standardized geometric model” overestimates the biomechanically measured stiffness for the young junctions of *O. ficus-indica* (Figure 3C). The overestimation of structural stiffness might have several reasons such as the scattering biomechanical data caused by biological variability. Furthermore, we can assume that simplification of the net-like arrangement of individual vascular bundles into a solid shell contributes to the overestimation, because initial forces in nets result mainly in re-alignment of strands before larger forces can be withstood by individual strands, whereas in a shell, initial forces directly lead to a deformation. We have refrained from adopting this net-like tissue layer of vascular bundles into our geometric models, as this would make the simulation more complex and would provide less value with regard to the knowledge gained for technical material systems. In order to align the simulation with the biomechanical data and to obtain an “adjusted geometric model,” we have iteratively reduced the shell thickness of the vascular bundles. The equipment of the shells of the “adjusted geometric models” with the respective mechanical properties of the older junction of *O. ficus-indica* and the junction of *C. bigelovii* leads, in each case, to an underestimation of the biomechanically measured values (Figure 4A). In the older samples of *O. ficus-indica*, we can assume that this is caused by the overall growth of the junction, and in the samples of *C. bigelovii*, by the presence of a larger relative proportion of dermal tissues and vascular bundles, although the junctions themselves are comparable in size.

The influence of the mechanical properties of the dermal tissues on the stiffness of the overall system depends strongly on the inner stiffness of the vascular bundles (Figure 5). *O. ficus-indica* junctions achieve a high effective tensile modulus even at a young stage, caused by the much higher elastic modulus of the vascular bundles (median 1,230 MPa) compared with the dermal tissues (median 44 MPa). Older junctions can modify the elastic modulus of dermal tissues by means of the periderm covering, which results in a median elastic modulus of 464 MPa. However, basal junctions with periderm accumulate additional layers, and the thickness of the dermal tissue has an increasingly larger influence on the overall stiffness. In addition, the elastic modulus of the vascular bundles in older *O. ficus-indica* junctions is increased to 1,670 MPa. Because of the very low elastic modulus of the vascular bundles in *C. bigelovii* (median 7 MPa), the stiffening of the dermal tissues has a much larger influence. Thus, the stiffness of the system can be kept low in lateral junctions, and high relative stiffening can be achieved in more basal junctions by periderm accumulation.

One aspect that has no influence on our tensile tests but might have a damping impact under (repetitive) bending load, especially at larger deflection, is the presence of trichomes that grow annularly around the young branch-branch junctions of both species (Figures 6A,C,E). In older junctions of *O. ficus-indica*, fewer trichomes are present and are mostly displaced by the periderm (Figures 6B,D).

### Fracture analysis

The fracture behavior of Opuntioideae can be interpreted as an adaptation to various propagation concepts, which are often associated with geometric properties and fracture mechanics of their lateral branch-branch junctions. In this study, we have compared *O. ficus-indica*, a species with predominantly sexual propagation by fruits/seeds, and *C. bigelovii*, a species known for its vegetative propagation by offshoots, with regard to their mechanical junction behavior under tensile loading.

Figures 9A–C show that the cactus junctions can be regarded as truncated components with notches. In general, a notch represents weakening of the structure, whereby notch effect depends on material properties and the type of load. In materials science, we distinguish between brittleness and ductility. Brittle materials fracture with little elastic deformation, without significant plastic and/or viscoelastic deformation, and with relatively small values of fracture energy but high strength. In contrast, ductile materials exhibit extensive plastic and/or viscoelastic deformation prior to fracture and high values of fracture energy. This classification into brittle and ductile materials cannot be readily applied to plant material systems, but commonalities can be seen in the fracture behavior. As in brittle materials, the young junctions of *O. ficus-indica* and the junctions of *C. bigelovii* (cf. Table 2 and Figures 6A,C) are particularly sensitive to the presence of notches and typically fail at stress concentrations (cf. Figure 9A). Like ductile materials, older junctions and branches of *O. ficus-indica* (cf. Table 2 and Figures 6D,F) mitigate stress peaks by viscoelastic plastic deformation or yielding, which allows further loading. In addition, areas near the notches (marked in gray in Figure 9B) are less stretched by the tensile load, an effect that is further enhanced by the stiff periderm (marked in red in Figure 9B). In Figure 9, we also provide information concerning maximum stress (=strength) and deformation at failure derived from tensile tests of the dermal tissues without and with periderm coverage, the strands of vascular bundles, and the entire junction. Unfortunately, we have been unable to perform experiments on the chlorenchyma and parenchyma. However, in the literature, we have found a value of 0.3 MPa for compressive strength (Gibson, 2012) and values ranging between 4 and 16% for deformation at break (Zdunek and Umeda, 2006). As with the elastic behavior, the fracture behavior of junctions is not simply a sum of the individual tissue properties. The decisive factors are the notch effect on a structure and the ability of a material system to mitigate the notch effect by viscoelastic plastic deformation.

Lateral branches of *C. bigelovii*, despite their comparable size and mass, fail at markedly lower median tensile forces (14 N) than the young (51 N) and the older (153 N) lateral junctions of *O. ficus-indica*. After normalization of the values over the junction area, the effect is even increased (Figures 7A–E) confirming the observation that *C. bigelovii* branches are especially prone to fail under slight mechanical (bending) loads (Bobich and Nobel, 2001a,b) and extends this finding to tensile forces. In nature, however, the combination of bending, tensile, torsional, and shear loads act on branches and their junctions. The advantage of mechanical analysis under tensile load is that only the cross-sectional area of the narrowest point has to be included for normalization of mechanical variables and not the complex change of axial, polar, or torsional second moments of area, as is the case with bending and torsion tests (Niklas, 1994). The branches of *O. ficus-indica* with their oval cross-sections that continually change along the longitudinal axis and the tapered branches of *C. bigelovii* with their irregular protrusions of the circular cross-section cannot be reliably determined, especially with respect to the variability of the junction samples.

The lower work of fracture and fracture energy values for *C. bigelovii*, together with the lower strain at *F*_*max*_ (Figures 7F–H) and junction strain at break values (Figure 7E) provide a mechanical explanation for the appearance of up to several hundred small plants in the direct vicinity of larger plants (Bobich and Nobel, 2001b), as the smaller plants have probably arisen by propagation from fallen branches. Because of strong slipping effects when testing markedly older *O. ficus-indica* junctions, our results are limited to the outermost branches. However, as Bobich and Nobel (2001a) have shown, chains of broken branches are also found in vegetatively propagating species, indicating that distinct increase in junction strength occurs only in most basal branches. In *O. ficus-indica*, the stiffening is not only evident in more basal junctions (Bobich and Nobel, 2001a,^2002^) but also occurs, as we have shown, in lateral junctions. This is reflected in the markedly higher values of force and work required for fracture. When normalized over the junction area, the obtained values are lower than those of the young branches (Figures 7D–G), a result that again can be explained by the allometric growth of the junctions and tissues involved. In addition, the differences in fracture modes between the young and old lateral junctions are changed markedly: the median value of the relative fracture range is increased more than ten-fold from the young (4.4%) to the older (47.2%) junctions. In addition, the number of samples with a smooth failure surface is decreased from thirteen to three (Supplementary Table 2). The failure of nine older samples of *O. ficus-indica* through the branch shows that the junctions are not the sole weak points of the samples and reveals the limits of the biomechanical testing capability of the junctions under tensile loading.

Even in *Opuntia* species known for their predominantly sexual reproduction, vegetative propagation *via* offshoots can also play a crucial role in the survival of the population (Grant and Grant, 1980; Mandujano et al., 1998, 2001). Mandujano et al. (2007) have shown that in *O. macrocentra*, clonal propagation occurs less frequently than sexual propagation, but that clones have a higher chance of survival, grow faster, and can reproduce more quickly. The rooting ability of detached *O. ficus-indica* branches (Gibson and Nobel, 1986) shows that vegetative propagation can also be found for this species. However, mechanical stiffening of the lateral junctions, as a result of fast branch growth and the accompanying formation of a (wound) peridermal collar around the areole from which the branch emerges (Mylo et al., 2021a), occurs even in young stages. This not only enhances the water storage capacity of the branches by increasing the amount of parenchyma tissue but at the same time the formation of the stiffening wound periderm makes the junctions less prone to mechanical failure.

### Deformation analysis

The junction strains of over 70% and the local surface strain concentration at and around the junction under tensile loading for all three tested groups illustrate the extent to which the junctions are the mechanical weak points of the Opuntioideae (Figure 8). The junction strain values of *O. ficus-indic*a, which are more than twice as high for both the young and the older junctions than for those of *C. bigelovii*, verify that they are less susceptible to failure than those of *C. bigelovii*. In addition, the strain values of the connected branch parts are also higher for *O. ficus-indica*, from which we can conclude that their connected branch parts are more extensively involved in the absorption of tensile stresses than those of *C. bigelovii*. The combination of these effects is also reflected in the significantly higher strains at *F*_*max*_ and fracture energy values for *O. ficus-indica* (Figures 7G,H).

The measured maximum strain rates of the entire sample, and especially that of the junction, are markedly higher than the maximum strain values of the dermal tissues (with and without the periderm covering) and vascular bundles involved (Mylo et al., 2021a). This suggests that additionally to the deformation of the tissues involved, other processes take place enabling larger total elongation. Reorganization of the loop-like notches and the net-like arranged vascular bundles in the lateral and basal branch parts might also contribute to the elongation, in addition to the change in geometry at the junction (a kind of bulging of internal tissues occurs at higher tensile loads). The latter effect might be another reason that the simplification of the vascular bundles as a shell (Figure 4) overestimates the mechanical properties of the entire system, making an approximately 80% reduction in the simulated shell thickness necessary in order to match the biomechanical data (Figure 3). However, the re-alignment of the vascular bundles under tensile loading is not visible from the outside and would require *in situ* tensile testing with simultaneous imaging (Hesse et al., 2019).

## Conclusion

We can provide the following answers to our three main scientific questions, which we repeat here.

(i) What are the elastic properties of the junctions under tensile loading and how can these be supported by finite element analyses? By tensile tests, we have shown that tensile stiffness is highest for the older junctions of *O. ficus-indica*, whereas that for the young branches of *O. ficus-indica* is about three times that of *C. bigelovii*, with the two groups not differing significantly in the weight of their lateral branches and junction area. After normalization of the data to the respective junction area, the significantly highest values have been found for the junctions with young branches in *O. ficus-indica*. The values from the older junctions of *O. ficus-indica* and those of *C. bigelovii* are lower by a factor of two to three, indicating the allometric growth of the tissues involved. The FE simulations have shown that the influence of a periderm covering around the junctions on the effective tensile moment is more than 10 factors higher in samples with vascular bundles having low Young’s modulus compared with samples with vascular bundles having high Young’s modulus.

(ii) What is the load-bearing capacity of the junctions and what are the fracture properties of the junctions under tensile loading? Most samples of the fracture-prone junctions of *C. bigelovii* abscise directly at the junction and exhibit cup and cone surfaces, and the relative fracture strain is on the median 28%. Most fracture-prone young samples of *O. ficus-indica* fail directly at the junction and have smooth fracture surfaces, and the relative fracture strain is on the median 4%. The majority of the fracture-resistant older samples of *O. ficus-indica* fail at the branch, exhibit rough fracture surfaces, and have, on median, a relative fracture strain of 47% of the total strain. The differences in the maximum force are of particular interest for the abscission of lateral branches triggered in nature by wind, passing animals, or vibration: 153 N (older *O. ficus-indica*), 51 N (young *O. ficus-indica*), and 14 N (*C. bigelovii*).

(iii) What influence does the growth of the lateral branches have on the mechanical properties of the junctions? In *C. bigelovii*, all the lateral junctions have been grouped together, and the normalized mechanical variables either show no dependence on the weight of the lateral branch (tensile strength and fracture energy) or the values are decreased with increase in weight (effective tensile modulus). In the young junctions of *O. ficus-indica*, we have found an increase with increase in apical branch weight for all the examined mechanical variables, indicating early mechanical stabilization of the junctions. In the older junctions, no dependence on the weight of the lateral branches has been determined for the normalized variables, or the dependence is slightly negative (tensile strength).

In summary, the results show that the lateral branches of *O. ficus-indica* are susceptible to fracture but stiffen at early stages by increased growth of the junction and formation of a periderm overlay, whereas these processes occur at a (much) slower rate in *C. bigelovii* or have not been found for lateral branches.

## Data availability statement

The original contributions presented in this study are included in the article/Supplementary material, further inquiries can be directed to the corresponding author/s.

## Author contributions

LP, TS, and OS acquired the funding for this study and supervised the research. MM, LP, TS, and OS planned and designed the research. MM constructed and printed the individualized clamps, conducted the biomechanical tensile tests with DIC, performed the statistical analysis, and wrote the first draft of the manuscript. MM and OS analyzed the biomechanical data. MM and AH constructed the geometric models. AH and LP conducted the FEM simulation. All authors critically discussed the data, contributed significantly to the final version of the manuscript, and gave their final approval.

## Supplementary material

Supplementary Figure 1 Fracture site comparison of older *Opuntia ficus-indica* junctions.

Supplementary Table 1 Properties of the finite element models.

Supplementary Table 2 Raw biomechanical data.

Supplementary Table 3 Detailed statistical analysis.

Supplementary Video 1 Local surface strain analysis of a young junction of *Opuntia ficus-indica*.

Supplementary Video 2 Local surface strain analysis of an older junction of *Opuntia ficus-indica*.

Supplementary Video 3 Local surface strain analysis of a junction of *Cylindropuntia bigelovii*.
