# Supplementary figures and images for: Elastic property and fracture mechanics of lateral branch-branch junctions in cacti: A case study of Opuntia ficus-indica and Cylindropuntia bigelovii
Source: Front Plant Sci. 2022 Sep 27;13:950860. doi: 10.3389/fpls.2022.950860 (PMC9551649; doi:10.3389/fpls.2022.950860)

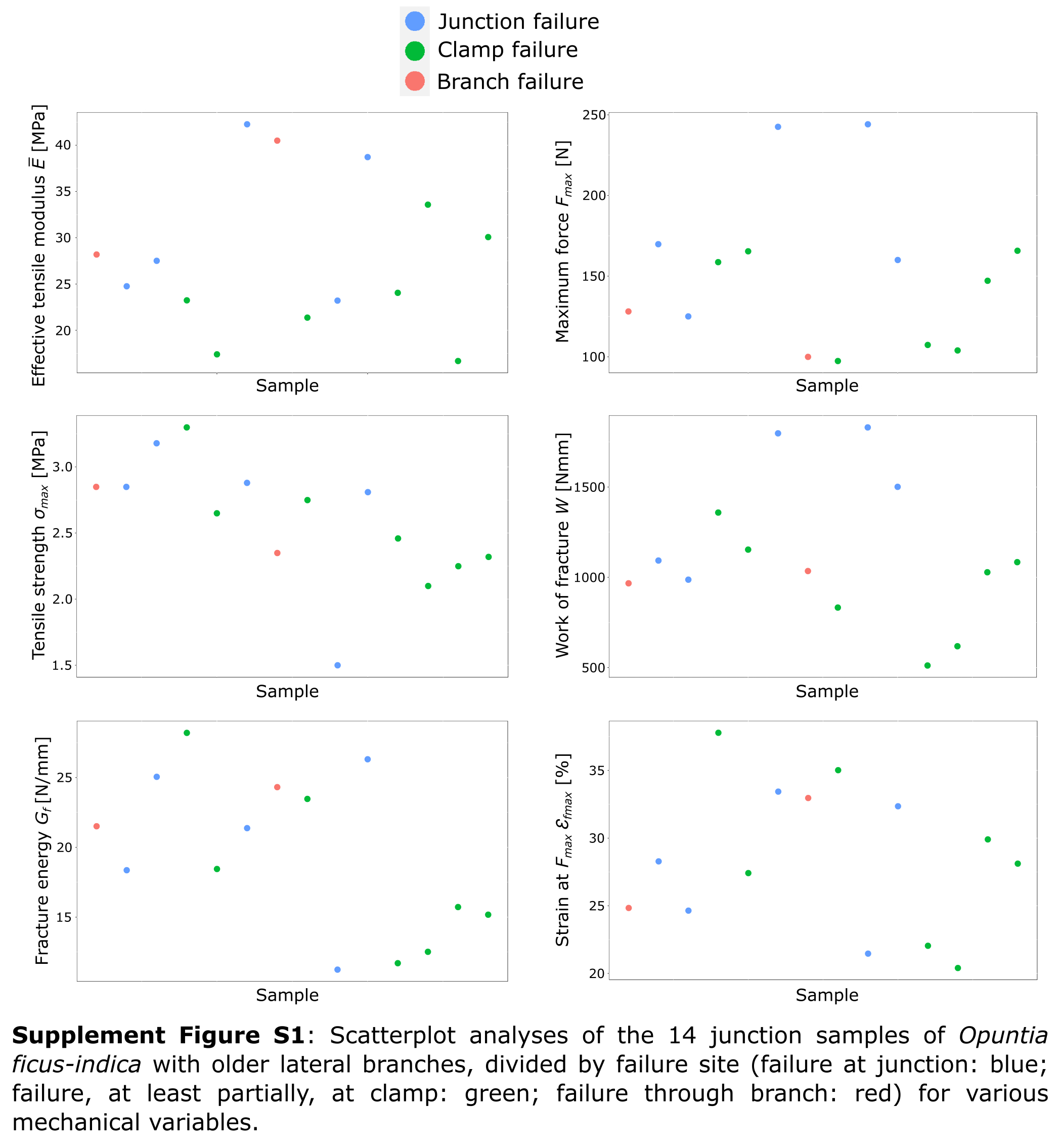

Supplement: Supplementary Figure 1 — Fracture site comparison of older Opuntia ficus-indica junctions. [file Image_1.jpeg]
