# Supplementary material for: Elastic property and fracture mechanics of lateral branch-branch junctions in cacti: A case study of Opuntia ficus-indica and Cylindropuntia bigelovii
Source: Front Plant Sci. 2022 Sep 27;13:950860. doi: 10.3389/fpls.2022.950860 (PMC9551649; doi:10.3389/fpls.2022.950860)
Supplement: Supplementary Table 1 — Properties of the finite element models. [file Data_Sheet_1.PDF]

Supplementary Table S1: Geometric and mechanical properties of the finite element models

Elastic properties and fracture mechanics of lateral branch-branch junctions in cacti: a case study of *Opuntia ficus-indica* and *Cylindropuntia bigelovii*

Max D. Mylo[1,2*], Anna Hoppe[2,3], Lars Pastewka[2,3], Thomas Speck[1,2], Olga Speck[1,2]

[1]Plant Biomechanics Group @ Botanic Garden Freiburg, University of Freiburg, Freiburg, Germany

[2]Cluster of Excellence *liv*MatS @ FIT – Freiburg Center for Interactive Materials and Bioinspired Technologies, Freiburg, Germany

[3]Department of Microsystems Engineering, University of Freiburg, Freiburg, Germany

***Correspondence:**

Max D. Mylo: max.mylo@biologie.uni-freiburg.de

Table S1. Geometric and mechanical properties of the finite element models. [1)] data derived from Mylo et al., 2021, [2)] data derived from Hesse et al. 2020.

FE models	Standardized geometric model	Adjusted geometric model	Opuntia_ model_young	Opuntia_ model_older	Cylindropuntia_ model
**Entire model**					
height	30 mm	30 mm	30 mm	30 mm	30 mm
**Shell of dermal tissues**					
Upper radii (oval cross-section)	11.5 mm, 4.5 mm	11.5 mm, 4.5 mm	11.5 mm, 4.5 mm	11.5 mm, 4.5 mm	11.5 mm, 4.5 mm
Junction radius (circular cross-section)	2.0 mm	2.0 mm	2.0 mm	2.0 mm	2.0 mm
Lower radii (oval cross-section)	21.0 mm, 4.5 mm	21.0 mm, 4.5 mm	21.0 mm, 4.5 mm	21.0 mm, 4.5 mm	21.0 mm, 4.5 mm
Thickness[1)]	0.22 mm	0.22 mm	0.22 mm	0.22 mm	0.22 mm
Elastic modulus[1)]	40 MPa	40 MPa	40 MPa	440 MPa	40 MPa
**Chlorenchyma shell**					
Upper radii (oval cross-section)	11.28 mm, 4.28 mm	11.28 mm, 4.28 mm	11.28 mm, 4.28 mm	11.28 mm, 4.28 mm	11.28 mm, 4.28 mm

Junction radius (circular cross-section)	1.78 mm	1.78 mm	1.78 mm	1.78 mm	1.78 mm
Lower radii (oval cross-section)	20.78 mm, 4.28 mm	20.78 mm, 4.28 mm	20.78 mm, 4.28 mm	20.78 mm, 4.28 mm	20.78 mm, 4.28 mm
Upper thickness	2.18 mm	2.18 mm	2.18 mm	2.18 mm	2.18 mm
Junction thickness	0.78 mm	0.78 mm	0.78 mm	0.78 mm	0.78 mm
Lower thickness	2.18 mm	2.18 mm	2.18 mm	2.18 mm	2.18 mm
Elastic modulus[2]	0.24 MPa	0.24 MPa	0.24 MPa	0.24 MPa	0.24 MPa
**Shell of vascular bundles**					
Upper radii (oval cross-section)	9.1 mm, 2.1 mm	9.1 mm, 2.1 mm	9.1 mm, 2.1 mm	9.1 mm, 2.1 mm	9.1 mm, 2.1 mm
Junction radius (circular cross-section)	1.0 mm	1.0 mm	1.0 mm	1.0 mm	1.0 mm
Lower radii (oval cross-section)	18.6 mm, 2.1 mm	18.6 mm, 2.1 mm	18.6 mm, 2.1 mm	18.6 mm, 2.1 mm	18.6 mm, 2.1 mm
Upper thickness	0.13 mm	0.24 mm	0.24 mm	0.24 mm	0.24 mm
Junction thickness	0.20 mm	0.039 mm	0.039 mm	0.039 mm	0.039 mm
Lower thickness	0.13 mm	0.24 mm	0.24 mm	0.24 mm	0.24 mm
Elastic modulus[1]	1200 MPa	1200 MPa	1200 MPa	1200 MPa	7.5 MPa
**Inner parenchyma shell**					
Upper radii (oval cross-section)	8.97 mm, 1.97 mm	9.076 mm, 2.076 mm	9.076 mm, 2.076 mm	9.076 mm, 2.076 mm	9.076 mm, 2.076 mm
Junction radius (circular cross-section)	0.8 mm	0.961 mm	0.961 mm	0.961 mm	0.961 mm
Lower radii (oval cross-section)	18.47 mm, 1.97 mm	18.576 mm, 2.076 mm	18.576 mm, 2.076 mm	18.576 mm, 2.076 mm	18.576 mm, 2.076 mm
Elastic modulus[2]	0.24 MPa	0.24 MPa	0.24 MPa	0.24 MPa	0.24 MPa

Literature cited

Hesse, L., Kampowski, T., Leupold, J., Caliaro, S., Speck, T., and Speck, O. (2020). Comparative analyses of the self-sealing mechanisms in leaves of *Delosperma cooperi* and *Delosperma ecklonis* (Aizoaceae). *Int. J. Mol. Sci*, 21, 5768. doi: 10.3390/ijms21165768

Mylo, M. D., Hesse, L., Masselter, T., Leupold, J., Drozella, K., Speck, T., and Speck, O. (2021). Morphology and Anatomy of Branch–Branch Junctions in *Opuntia ficus-indica* and *Cylindropuntia bigelovii*: A Comparative Study Supported by Mechanical Tissue Quantification. *Plants* 10, 2313. doi: 10.3390/plants10112313
