# Supplementary material for: Elastic property and fracture mechanics of lateral branch-branch junctions in cacti: A case study of Opuntia ficus-indica and Cylindropuntia bigelovii
Source: Front Plant Sci. 2022 Sep 27;13:950860. doi: 10.3389/fpls.2022.950860 (PMC9551649; doi:10.3389/fpls.2022.950860)
Supplement: Supplementary Table 2 — Raw biomechanical data. [file Data_Sheet_2.PDF]

Supplementary Table 2: Geometric, physical, and biomechanical raw data of cacti junctions

Elastic properties and fracture mechanics of lateral branch-branch junctions in cacti: a case study on *Opuntia ficus-indica* and *Cylindropuntia bigelovii*

Opuntia ficus indica (young lateral junction)

Sample	Plant	Weight [g] apical untested	Weight [g] apical tested	Weight [g] total	Fracture surface	Fracture site	Junction area Basal [mm²]	Junction area Apical [mm²]	Junction area Mean [mm²]	Junction area Ratio	Maximum Force [N]	Maximum Strength [MPa]	Tensile Stiffness [N/mm]	Elastic Modulus [MPa]	Work of Fracture [mJ]	Fracture Energy [mJ/mm²]	Strain at *Fmax* [%]	Relative elastic range [%]	Relative plastic range [%]	Relative fracture range [%]
**01**	A	4.3	9.8	**14.1**	smooth	junction	7.75	7.63	**7.69**	1.016	**29.91**	**3.89**	**5.22**	**20.37**	**86.78**	**11.28**	**20.82**	**89.99**	**6.27**	**3.74**
**02**	A	21.6	12.9	**34.5**	smooth	junction	12.42	12.72	**12.57**	0.976	**60.21**	**4.79**	**23.55**	**56.19**	**271.37**	**21.59**	**21.87**	**21.41**	**72.14**	**6.45**
**03**	A	3.1	6.2	**9.3**	smooth	junction	8.32	8.68	**8.50**	0.959	**25.67**	**3.02**	**11.65**	**41.12**	**75.45**	**8.88**	**13.47**	**15.25**	**75.33**	**9.42**
**04**	A	12.9	9.8	**22.7**	smooth	junction	12.78	12.93	**12.86**	0.988	**41.66**	**3.24**	**15.63**	**36.48**	**192.55**	**14.98**	**22.08**	**23.71**	**64.69**	**11.60**
**05**	A	12.7	10.5	**23.2**	smooth	junction	14.53	14.99	**14.76**	0.969	**53.12**	**3.60**	**20.88**	**42.43**	**266.04**	**18.02**	**25.43**	**18.95**	**79.51**	**1.54**
**06**	A	4.6	8.0	**12.6**	smooth	junction	7.23	7.53	**7.38**	0.960	**27.94**	**3.79**	**16.22**	**65.95**	**81.67**	**11.07**	**14.47**	**23.21**	**73.68**	**3.11**
**07**	A	12.0	10.5	**22.5**	smooth	junction	16.17	16.14	**16.16**	1.002	**54.41**	**3.37**	**17.06**	**31.68**	**292.21**	**18.09**	**27.12**	**22.91**	**71.61**	**5.48**
**08**	B	2.5	7.2	**9.7**	smooth	junction	5.41	5.34	**5.37**	1.012	**21.07**	**3.92**	**8.44**	**47.10**	**46.21**	**8.60**	**12.94**	**68.21**	**26.15**	**5.64**
**09**	B	13.4	9.8	**23.2**	smooth	junction	13.04	13.68	**13.36**	0.953	**53.57**	**4.01**	**25.86**	**58.06**	**313.86**	**23.49**	**27.14**	**16.86**	**78.46**	**4.68**
**10**	B	6.0	8.2	**14.2**	smooth	junction	8.21	8.76	**8.49**	0.937	**31.51**	**3.71**	**3.40**	**12.01**	**233.21**	**26.30**	**42.94**	**57.06**	**40.81**	**2.12**
**11**	B	18.1	11.2	**29.3**	smooth	junction	13.14	12.51	**12.83**	1.050	**55.81**	**4.35**	**24.90**	**58.26**	**304.77**	**23.76**	**25.34**	**17.45**	**78.51**	**4.04**
**12**	B	15.4	10.3	**25.7**	smooth	junction	20.03	20.42	**20.23**	0.981	**70.16**	**3.47**	**29.54**	**43.82**	**371.39**	**18.36**	**24.74**	**17.10**	**78.16**	**4.74**
**13**	B	15.8	10.8	**26.6**	smooth	junction	12.04	11.70	**11.87**	1.029	**49.38**	**4.16**	**24.69**	**62.39**	**235.43**	**19.83**	**25.47**	**18.79**	**79.64**	**1.57**
**14**	B	19.7	12.5	**32.2**	cup & cone	junction & branch	13.52	13.62	**13.57**	0.993	**77.09**	**5.68**	**30.15**	**66.65**	**461.48**	**34.01**	**27.36**	**13.19**	**82.83**	**3.98**
**Min**				**9.3**					**5.37**	0.937	**21.07**	**3.02**	**3.40**	**12.01**	**46.21**	**8.60**	**12.94**	**13.19**	**6.27**	**1.54**
**Max**				**34.5**					**20.23**	1.050	**77.09**	**5.68**	**30.15**	**66.65**	**461.48**	**34.01**	**42.94**	**89.99**	**82.83**	**11.60**
**Mean**				**21.4**					**11.83**	0.988	**46.54**	**3.93**	**18.37**	**45.89**	**230.89**	**18.45**	**23.66**	**30.29**	**64.84**	**4.87**
**SD**				**7.9**					**3.84**	0.031	**16.65**	**0.66**	**8.41**	**16.13**	**117.92**	**6.95**	**7.26**	**22.75**	**22.52**	**2.74**
**Median**				**23.0**					**12.70**	0.985	**51.25**	**3.84**	**18.97**	**45.46**	**250.74**	**18.23**	**25.04**	**20.18**	**74.51**	**4.36**
**IQR**				**12.25**					**5.03**	0.047	**25.15**	**0.62**	**12.20**	**20.57**	**188.41**	**10.81**	**5.62**	**6.39**	**12.08**	**2.33**

©Plant Biomechanics Group Freiburg - Mylo et al. 2022

Opuntia ficus indica (older lateral junction)

Sample	Plant	Weight [g] apical untested	Weight [g] apical tested	Weight [g] total	Fracture surface	Fracture site	Junction area Basal [mm²]	Junction area Apical [mm²]	Junction area Mean [mm²]	Ratio	Maximum Force [N]	Maximum Strength [MPa]	Tensile Stiffness [N/mm]	Elastic Modulus [MPa]	Work of Fracture [mJ]	Fracture Energy [mJ/mm²]	Strain at *Fmax* [%]	Relative elastic range [%]	Relative plastic range [%]	Relative fracture range [%]
**01**	C	16.9	21.4	**38.3**	rough	branch	45.19	44.67	**44.93**	1.012	**128.15**	**2.85**	**41.78**	**28.42**	**966.87**	**21.52**	**24.84**	**14.60**	**43.39**	**42.01**
**02**	C	18.0	21.9	**39.9**	cup & cone	junction & branch	43.22	41.89	**42.55**	1.032	**100.00**	**2.35**	**13.18**	**10.27**	**1034.72**	**24.32**	**46.30**	**42.02**	**6.22**	**51.76**
**03**	C	30.8	21.2	**52.0**	cup & cone	junction & branch	47.33	49.03	**48.18**	0.965	**158.75**	**3.30**	**32.09**	**18.67**	**1358.98**	**28.21**	**37.79**	**12.54**	**24.63**	**62.83**
**04**	C	17.2	18.4	**35.6**	rough	branch	60.72	64.32	**62.52**	0.944	**165.44**	**2.65**	**38.29**	**18.17**	**1153.44**	**18.45**	**27.42**	**1.86**	**55.02**	**43.12**
**05**	C	13.5	18.5	**32.0**	rough	branch	35.40	35.54	**35.47**	0.996	**97.40**	**2.75**	**34.86**	**24.00**	**832.46**	**23.47**	**35.02**	**3.57**	**46.26**	**50.17**
**06**	C	15.9	22.4	**38.3**	smooth	junction	42.65	44.79	**43.72**	0.952	**107.36**	**2.46**	**32.63**	**23.46**	**511.54**	**11.70**	**22.05**	**29.72**	**55.59**	**14.69**
**07**	C	15.6	20.4	**36.0**	rough	branch	50.66	48.12	**49.39**	1.053	**103.95**	**2.10**	**55.74**	**33.36**	**618.41**	**12.52**	**20.40**	**12.73**	**28.87**	**58.39**
**08**	D	15.6	22.2	**37.8**	rough	branch	67.15	63.57	**65.36**	1.056	**147.19**	**2.25**	**17.27**	**7.41**	**1028.13**	**15.73**	**29.91**	**28.34**	**12.09**	**59.57**
**09**	D	27.2	23.6	**50.8**	smooth	junction	70.40	72.49	**71.45**	0.971	**165.76**	**2.32**	**72.81**	**28.53**	**1084.27**	**15.18**	**28.12**	**4.83**	**68.38**	**26.80**
**10**	D	24.0	17.2	**41.2**	smooth	junction	58.75	60.27	**59.51**	0.975	**169.89**	**2.85**	**59.13**	**29.70**	**1093.30**	**18.37**	**28.28**	**10.84**	**81.71**	**7.45**
**11**	D	21.4	17.7	**39.1**	rough	branch	38.68	40.10	**39.39**	0.965	**125.12**	**3.18**	**42.24**	**32.34**	**987.28**	**25.06**	**24.64**	**2.48**	**39.37**	**58.15**
**12**	D	46.6	22.0	**68.6**	rough	branch	87.52	80.69	**84.11**	1.085	**242.63**	**2.88**	**39.02**	**13.92**	**1797.78**	**21.38**	**33.44**	**43.28**	**14.01**	**42.72**
**13**	D	52.1	32.3	**84.4**	rough	branch	169.42	156.41	**162.92**	1.083	**244.23**	**1.50**	**106.50**	**22.32**	**1830.74**	**11.24**	**21.47**	**3.45**	**48.56**	**47.99**
**14**	D	16.5	19.6	**36.1**	rough	branch	57.39	56.70	**57.05**	1.012	**160.09**	**2.81**	**64.22**	**32.82**	**1501.52**	**26.32**	**32.36**	**4.58**	**48.92**	**46.49**
**Min**				**32.0**					**35.47**	0.944	**97.40**	**1.50**	**13.18**	**7.41**	**511.54**	**11.24**	**20.40**	**1.86**	**6.22**	**7.45**
**Max**				**84.4**					**162.92**	1.085	**244.23**	**3.30**	**106.50**	**33.36**	**1830.74**	**28.21**	**46.30**	**43.28**	**81.71**	**62.83**
**Mean**				**45.0**					**61.90**	1.007	**151.14**	**2.59**	**46.41**	**23.10**	**1128.53**	**19.53**	**29.43**	**15.35**	**40.93**	**43.72**
**SD**				**14.2**					**30.91**	0.046	**45.36**	**0.45**	**23.17**	**8.14**	**371.35**	**5.43**	**6.87**	**14.03**	**20.98**	**16.08**
**Median**				**38.7**					**53.22**	1.004	**152.97**	**2.70**	**40.40**	**23.73**	**1059.50**	**19.91**	**28.20**	**11.69**	**44.82**	**47.24**
**IQR**				**11.88**					**20.63**	0.081	**53.88**	**0.53**	**25.10**	**11.11**	**335.63**	**8.79**	**8.48**	**21.09**	**27.80**	**14.37**

©Plant Biomechanics Group Freiburg - Mylo et al. 2022

Cylindropuntia bigelovii

Sample	Plant	Weight [g] untested	Weight [g] apical tested	Weight [g] total	Fracture surface	Fracture site	Junction area Basal [mm²]	Junction area Apical [mm²]	Junction area Mean [mm²]	Ratio	Maximum Force [N]	Maximum Strength [MPa]	Tensile Stiffness [N/mm]	Elastic Modulus [MPa]	Work of Fracture [mJ]	Fracture Energy [mJ/mm²]	Strain at *Fmax* [%]	Relative elastic range [%]	Relative plastic range [%]	Relative fracture range [%]
**01**	A	5.4	7.5	**12.9**	cup & cone	junction & branch	8.86	9.18	**9.02**	0.966	**11.29**	**1.25**	**5.26**	**17.49**	**29.35**	**3.26**	**11.11**	**41.55**	**28.32**	**30.14**
**02**	A	1.6	6.6	**8.2**	cup & cone	junction & branch	6.86	6.67	**6.76**	1.029	**8.68**	**1.28**	**3.33**	**14.79**	**25.57**	**3.78**	**14.31**	**34.77**	**22.85**	**42.38**
**03**	A	13.7	9.7	**23.4**	smooth	junction	13.36	13.67	**13.51**	0.977	**18.58**	**1.38**	**8.62**	**19.14**	**30.90**	**2.29**	**9.38**	**49.29**	**42.96**	**7.75**
**04**	A	10.7	7.6	**18.3**	cup & cone	junction & branch	11.61	11.66	**11.63**	0.996	**17.90**	**1.54**	**6.84**	**17.63**	**60.37**	**5.19**	**16.24**	**8.41**	**78.77**	**12.82**
**05**	A	6.1	6.5	**12.6**	cup & cone	junction & branch	9.79	10.79	**10.29**	0.907	**15.08**	**1.47**	**5.21**	**15.18**	**48.68**	**4.73**	**16.37**	**17.11**	**47.32**	**35.57**
**06**	A	0.0	2.5	**2.5**	smooth	junction	5.24	5.04	**5.14**	1.039	**6.60**	**1.28**	**5.40**	**31.50**	**9.67**	**1.88**	**7.31**	**19.10**	**63.51**	**17.39**
**07**	A	9.8	4.6	**14.4**	cup & cone	junction & branch		11.42	**11.42**		**17.37**	**1.52**	**5.22**	**13.72**	**84.59**	**7.41**	**16.37**	**19.29**	**11.34**	**69.37**
**08**	A	4.0	6.7	**10.7**	smooth	junction	9.03	9.00	**9.01**	1.003	**14.25**	**1.58**	**7.28**	**24.21**	**19.33**	**2.14**	**8.77**	**25.63**	**70.26**	**4.12**
**09**	A	17.7	9.1	**26.8**	cup & cone	junction & branch		18.07	**18.07**		**14.41**	**0.80**	**7.85**	**13.04**	**44.56**	**2.47**	**11.78**	**6.99**	**43.15**	**49.86**
**10**	A	4.7	8.2	**12.9**	cup & cone	junction & branch	9.99	10.47	**10.23**	0.954	**13.91**	**1.36**	**8.71**	**25.53**	**41.45**	**4.05**	**12.31**	**7.13**	**66.05**	**26.82**
**11**	A	2.4	7.5	**9.9**	cup & cone	junction & branch		10.05	**10.05**		**14.14**	**1.41**	**5.29**	**15.78**	**47.50**	**4.73**	**16.11**	**13.14**	**59.62**	**27.24**
**12**	A	15.3	11.7	**27.0**	cup & cone	junction & branch	16.81	17.06	**16.94**	0.986	**24.07**	**1.42**	**6.41**	**11.35**	**91.03**	**5.38**	**17.31**	**15.83**	**62.01**	**22.16**
**13**	A	14.0	9.9	**23.9**	cup & cone	junction & branch	11.71	12.70	**12.21**	0.922	**18.08**	**1.48**	**6.32**	**15.53**	**54.69**	**4.48**	**12.97**	**13.04**	**47.17**	**39.80**
**14**	A	4.7	8.3	**13.0**	cup & cone	junction & branch	12.21	12.01	**12.11**	1.017	**13.53**	**1.12**	**6.33**	**15.69**	**37.04**	**3.06**	**12.04**	**18.82**	**49.03**	**32.15**
**15**	A	10.6	10.4	**21.0**	cup & cone	junction & branch	11.21	11.33	**11.27**	0.989	**20.96**	**1.86**	**8.72**	**23.21**	**48.19**	**4.28**	**12.57**	**15.16**	**57.38**	**27.46**
**Min**				**2.5**					**5.14**	0.907	**6.60**	**0.80**	**3.33**	**11.35**	**9.67**	**1.88**	**7.31**	**6.99**	**11.34**	**4.12**
**Max**				**27.0**					**18.07**	1.039	**24.07**	**1.86**	**8.72**	**31.50**	**91.03**	**7.41**	**17.31**	**49.29**	**78.77**	**69.37**
**Mean**				**15.8**					**11.18**	0.982	**15.26**	**1.38**	**6.45**	**18.25**	**44.86**	**3.94**	**13.00**	**20.35**	**49.98**	**29.67**
**SD**				**7.0**					**3.23**	0.038	**4.34**	**0.23**	**1.51**	**5.34**	**21.37**	**1.44**	**2.98**	**12.07**	**17.86**	**16.19**
**Median**				**13.0**					**11.27**	0.988	**14.41**	**1.41**	**6.33**	**15.78**	**44.56**	**4.05**	**12.57**	**17.11**	**49.03**	**27.46**
**IQR**				**10.6**					**2.62**	0.044	**4.27**	**0.22**	**2.29**	**6.19**	**21.56**	**1.97**	**4.73**	**9.37**	**19.71**	**17.91**
**Fallen branch**	A			**8.2**																
**Fallen branch**	A			**7.3**																
**Fallen branch**	A			**8.9**				4.6	**4.60**											

©Plant Biomechanics Group Freiburg - Mylo et al. 2022
