# Supplementary material for: Elastic property and fracture mechanics of lateral branch-branch junctions in cacti: A case study of Opuntia ficus-indica and Cylindropuntia bigelovii
Source: Front Plant Sci. 2022 Sep 27;13:950860. doi: 10.3389/fpls.2022.950860 (PMC9551649; doi:10.3389/fpls.2022.950860)
Supplement: Supplementary Table 3 — Detailed statistical analysis. [file Data_Sheet_3.PDF]

# Statistical analysis

## Junction area

| Species | Opuntia ficus-indica (young) | | | Opuntia ficus-indica (older) | | | Cylindropuntia bigelovii |
|---|---|---|---|---|---|---|---|
| **Group** | **Plant A** | **Plant B** | **Pooled** | **Plant A** | **Plant B** | **Pooled** | |
| **Normal distriburtion Shapiro test (p-value)** | 0.2928 | 0.6609 | 0.6356 | 0.4811 | 0.0237 | 0.0003 | 0.5242 |
| **Differences between plants Kruskal-Wallis test with Dunn post-hoc (p-value)** | 1.0000 | | | 1.0000 | | | |
| **Differences between groups Kruskal-Wallis test with Dunn post-hoc (p-value)** | **O. ficus-indica (young) vs. O ficus-indica (old)** | | | **O. ficus-indica (young) vs. C. bigelovii** | | | **O. ficus-indica (old) vs. C. bigelovii** |
| | < 0.0001 | | | 0.6358 | | | < 0.0001 |

## Apical branch weight

| Species | | Opuntia ficus-indica (young) | | | Opuntia ficus-indica (older) | | | Cylindropuntia bigelovii |
|---|---|---|---|---|---|---|---|---|
| **Group** | | **Plant A** | **Plant B** | **Pooled** | **Plant A** | **Plant B** | **Pooled** | |
| **Normal distriburtion Shapiro test (p-value)** | | 0.5104 | 0.4104 | 0.3596 | 0.0739 | 0.0636 | 0.0009 | 0.4105 |
| **Equality of variances (plants) Levene test (p-value)** | 0.2338 | | | | | | | |
| **Differences between plants ANOVA with TukeyHSD post-hoc (p-value)** | | 0.9661 | | | 0.1748 | | | |
| **Differences between groups Kruskal-Wallis test with Dunn post-hoc (p-value)** | | **O. ficus-indica (young) vs. O ficus-indica (old)** | | | **O. ficus-indica (young) vs. C. bigelovii** | | | **O. ficus-indica (old) vs. C. bigelovii** |
| | | 0.0002 | | | 0.2929 | | | < 0.0001 |

## Maximum force

| Species | | Opuntia ficus-indica (young) | | | Opuntia ficus-indica (older) | | | Cylindropuntia bigelovii |
|---|---|---|---|---|---|---|---|---|
| **Group** | | **Plant A** | **Plant B** | **Pooled** | **Plant A** | **Plant B** | **Pooled** | |
| **Normal distriburtion Shapiro test (p-value)** | | 0.2406 | 0.8093 | 0.4869 | 0.0737 | 0.1261 | 0.0494 | 0.9216 |
| **Equality of variances (plants) Levene test (p-value)** | 0.0144 | | | | | | | |
| **Differences between plants Kruskal-Wallis test with Dunn post-hoc (p-value)** | | 0.7657 | | | 0.8619 | | | |
| **Differences between groups Kruskal-Wallis test with Dunn post-hoc (p-value)** | | **O. ficus-indica (young) vs. O ficus-indica (old)** | | | **O. ficus-indica (young) vs. C. bigelovii** | | | **O. ficus-indica (old) vs. C. bigelovii** |
| | | 0.0030 | | | 0.0042 | | | < 0.0001 |

**©Plant Biomechanics Group Freiburg - Mylo et al. 2022**

## Maximum strength

| Species | | Opuntia ficus-indica (young) | | | Opuntia ficus-indica (older) | | | Cylindropuntia bigelovii |
|---|---|---|---|---|---|---|---|---|
| Group | | Plant A | Plant B | Pooled | Plant A | Plant B | Pooled | |
| Normal distriburtion Shapiro test (p-value) | | 0.4249 | 0.0819 | 0.1549 | 0.9692 | 0.3453 | 0.5859 | 0.3465 |
| Equality of variances (plants) Levene test (p-value) | 0.2620 | | | | | | | |
| Equality of variances (groups) Levene test (p-value) | 0.0555 | | | | | | | |
| Differences between plants ANOVA with TukeyHSD post-hoc (p-value) | | | 0.6509 | | | 0.9964 | | |
| Differences between groups ANOVA with TukeyHSD post-hoc (p-value) | | | **O. ficus-indica (young) vs. O ficus-indica (old)** | | | **O. ficus-indica (young) vs. C. bigelovii** | | **O. ficus-indica (old) vs. C. bigelovii** |
| | | | < 0.0001 | | | < 0.0001 | | < 0.0001 |

## Tensile stiffness

| Species | | Opuntia ficus-indica (young) | | | Opuntia ficus-indica (older) | | | Cylindropuntia bigelovii |
|---|---|---|---|---|---|---|---|---|
| Group | | Plant A | Plant B | Pooled | Plant A | Plant B | Pooled | |
| Normal distriburtion Shapiro test (p-value) | | 0.8110 | 0.0385 | 0.4438 | 0.6246 | 0.9306 | 0.2046 | 0.2936 |
| Equality of variances (groups) Levene test (p-value) | 0.0011 | | | | | | | |
| Differences between plants ANOVA with TukeyHSD post-hoc (p-value) | | | 0.6396 | | | 0.9385 | | |
| Differences between groups Kruskal-Wallis test with Dunn post-hoc (p-value) | | | **O. ficus-indica (young) vs. O ficus-indica (old)** | | | **O. ficus-indica (young) vs. C. bigelovii** | | **O. ficus-indica (old) vs. C. bigelovii** |
| | | | 0.0077 | | | 0.0131 | | < 0.0001 |

## Tensile modulus

| Species | | Opuntia ficus-indica (young) | | | Opuntia ficus-indica (older) | | | Cylindropuntia bigelovii |
|---|---|---|---|---|---|---|---|---|
| Group | | Plant A | Plant B | Pooled | Plant A | Plant B | Pooled | |
| Normal distriburtion Shapiro test (p-value) | | 0.9244 | 0.0777 | 0.4004 | 0.9693 | 0.1836 | 0.3261 | 0.0683 |
| Equality of variances (plants) Levene test (p-value) | 0.0580 | | | | | | | |
| Equality of variances (groups) Levene test (p-value) | 0.0015 | | | | | | | |
| Differences between plants ANOVA or Kruskal test with post-hoc (p-value) | | | 0.9730 | | | 0.9991 | | |
| Differences between groups ANOVA or Kruskal test with post-hoc (p-value) | | | **O. ficus-indica (young) vs. O ficus-indica (old)** | | | **O. ficus-indica (young) vs. C. bigelovii** | | **O. ficus-indica (old) vs. C. bigelovii** |
| | | | 0.0067 | | | 0.0001 | | 0.1957 |

©Plant Biomechanics Group Freiburg - Mylo et al. 2022

## Work of fracture

| Species | | Opuntia ficus-indica (young) | | | Opuntia ficus-indica (older) | | | Cylindropuntia bigelovii |
|---|---|---|---|---|---|---|---|---|
| Group | | Plant A | Plant B | Pooled | Plant A | Plant B | Pooled | |
| Normal distriburtion Shapiro test (p-value) | | 0.0660 | 0.7629 | 0.4595 | 0.9527 | 0.0531 | 0.3943 | 0.4884 |
| Equality of variances (plants) Levene test (p-value) | 0.0024 | | | | | | | |
| Equality of variances (groups) Levene test (p-value) | 0.0003 | | | | | | | |
| Differences between plants Kruskal-Wallis test with Dunn post-hoc (p-value) | | | 0.6549 | | | 1.0000 | | |
| Differences between groups Kruskal-Wallis test with Dunn post-hoc (p-value) | | | O. ficus-indica (young) vs. O ficus-indica (old) | | | O. ficus-indica (young) vs. C. bigelovii | | O. ficus-indica (old) vs. C. bigelovii |
| | | | 0.0035 | | | 0.0059 | | < 0.0001 |

## Fracture energy

| Species | | Opuntia ficus-indica (young) | | | Opuntia ficus-indica (older) | | | Cylindropuntia bigelovii |
|---|---|---|---|---|---|---|---|---|
| Group | | Plant A | Plant B | Pooled | Plant A | Plant B | Pooled | |
| Normal distriburtion Shapiro test (p-value) | | 0.6402 | 0.8700 | 0.6342 | 0.5739 | 0.7866 | 0.4844 | 0.5075 |
| Equality of variances (plants) Levene test (p-value) | 0.0135 | | | | | | | |
| Equality of variances (groups) Levene test (p-value) | 0.0008 | | | | | | | |
| Differences between plants Kruskal-Wallis test with Dunn post-hoc (p-value) | | | 0.9605 | | | 1.0000 | | |
| Differences between groups Kruskal-Wallis test with Dunn post-hoc (p-value) | | | O. ficus-indica (young) vs. O ficus-indica (old) | | | O. ficus-indica (young) vs. C. bigelovii | | O. ficus-indica (old) vs. C. bigelovii |
| | | | 0.6088 | | | < 0.0001 | | < 0.0001 |

## Strain at *Fmax*

| Species | | Opuntia ficus-indica (young) | | | Opuntia ficus-indica (older) | | | Cylindropuntia bigelovii |
|---|---|---|---|---|---|---|---|---|
| Group | | Plant A | Plant B | Pooled | Plant A | Plant B | Pooled | |
| Normal distriburtion Shapiro test (p-value) | | 0.4123 | 0.1123 | 0.0414 | 0.5313 | 0.7927 | 0.3970 | 0.3732 |
| Equality of variances (plants) Levene test (p-value) | 0.0135 | | | | | | | |
| Differences between plants Kruskal-Wallis test with Dunn post-hoc (p-value) | | | 1.0000 | | | 0.9321 | | |
| Differences between groups Kruskal-Wallis test with Dunn post-hoc (p-value) | | | O. ficus-indica (young) vs. O ficus-indica (old) | | | O. ficus-indica (young) vs. C. bigelovii | | O. ficus-indica (old) vs. C. bigelovii |
| | | | 0.1041 | | | 0.0014 | | < 0.0001 |

©Plant Biomechanics Group Freiburg - Mylo et al. 2022
